# Regulating Bacterial Behavior within Hydrogels of Tunable Viscoelasticity

**DOI:** 10.1002/advs.202106026

**Published:** 2022-04-11

**Authors:** Shardul Bhusari, Shrikrishnan Sankaran, Aránzazu del Campo

**Affiliations:** ^1^ INM ‐ Leibniz Institute for New Materials Campus D2 2 66123 Saarbrücken Germany; ^2^ Chemistry Department Saarland University 66123 Saarbrücken Germany

**Keywords:** bacterial hydrogel, bacterial–materials interactions, cell encapsulation, dynamic hydrogel, engineered living material

## Abstract

Engineered living materials (ELMs) are a new class of materials in which living organism incorporated into diffusive matrices uptake a fundamental role in material's composition and function. Understanding how the spatial confinement in 3D can regulate the behavior of the embedded cells is crucial to design and predict ELM's function, minimize their environmental impact and facilitate their translation into applied materials. This study investigates the growth and metabolic activity of bacteria within an associative hydrogel network (Pluronic‐based) with mechanical properties that can be tuned by introducing a variable degree of acrylate crosslinks. Individual bacteria distributed in the hydrogel matrix at low density form functional colonies whose size is controlled by the extent of permanent crosslinks. With increasing stiffness and elastic response to deformation of the matrix, a decrease in colony volumes and an increase in their sphericity are observed. Protein production follows a different pattern with higher production yields occurring in networks with intermediate permanent crosslinking degrees. These results demonstrate that matrix design can be used to control and regulate the composition and function of ELMs containing microorganisms. Interestingly, design parameters for matrices to regulate bacteria behavior show similarities to those elucidated for 3D culture of mammalian cells.

## Graphical abstract

Understanding bacterial behavior in confinement is of great interest for designing engineered living materials or studying biofilms. In this work, a unique hydrogel platform, whose viscoelastic properties can be tuned by varying the extent of stabilizing crosslinks, is developed to demonstrate that the growth and metabolism of encapsulated bacteria can be regulated by mechanical restrictions imparted by the matrix.

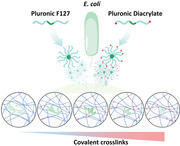

## 1 Introduction

The combination of synthetic biology and materials science has given rise to the field of engineered living materials (ELMs), wherein live organisms (bacteria, yeast, algae, etc.) become active components of material's design and perform advanced functions.^[^ ^1^, ^2^, ^3^ ^]^ Examples of ELMs include biofilters to sequester metals^[^ ^4^ ^]^ or viruses,^[^ ^5^ ^]^ bacterial hydrogels for biosensing,^[^ ^6^ ^]^ shape‐morphing composites,^[^ ^7^ ^]^ self‐healing adhesives,^[^ ^8^ ^]^ photosynthetic biogarments^[^ ^9^ ^]^ or self‐regulated drug delivery devices.^[^ ^10^ ^]^ A common feature in these constructs is the encapsulation of the organisms within matrices including natural polymers like agarose,^[^ ^11^, ^12^ ^]^ alginate,^[^ ^13^ ^]^ and dextran,^[^ ^14^ ^]^ synthetic polymers like polyvinyl alcohol^[^ ^15^ ^]^ and Pluronic,^[^ ^16^, ^17^ ^]^ or inorganic matrices like porous silica.^[^ ^18^ ^]^ Alternatively, proteinaceous^[^ ^19^ ^]^ or cellulose^[^ ^6^ ^]^ matrices produced by the organisms themselves, as in a biofilm, can serve as encapsulating networks. The matrix confers a protective environment for the cells while it allows the diffusion of nutrients and gases to maintain the viability and functionality of the entrapped organisms. It also confines and retains the organisms inside the material, which is a necessary requirement in the future application of ELMs containing genetically modified organisms.

**Figure 1:**
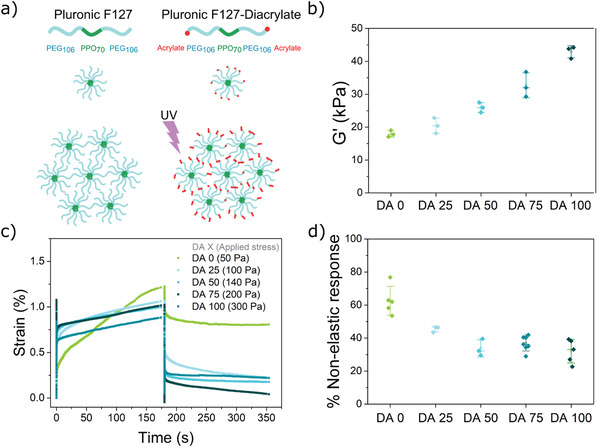
Structure and properties of Plu/PluDA hydrogels (DA 0‐100). a) The amphiphilic Pluronic F127 chains form micellar assemblies above 14 °C. Acrylate terminated Pluronic‐DA introduces covalent inter and intramicellar crosslinks after the photoinitiated radical polymerization step in the presence of Irgacure 2959. b) Storage modulus of Plu/PluDA hydrogels with increasing PluDA concentration after photoinitiated polymerization. c) Representative creep/recovery curves of Plu/PluDA hydrogels to reach a peak strain of ≈1% in 3 min. The stress applied to each hydrogel is indicated in the legend. d) Percentage of nonelastic response calculated from the creep experiment in c). (*N* ≥ 3, whiskers indicate standard deviation values and horizontal line in the middle denote median).

Recent studies have highlighted that spatial confinement and matrix mechanical properties affect the growth and functionality of embedded bacteria in ELMs.^[^ ^10^, ^20^, ^21^ ^]^ The current understanding, mainly from studies of bacteria or yeast embedded in hydrogels, indicate that the microbes grow inside the hydrogel network to form dense clusters at slower rates than in suspension.^[^ ^20^, ^22^, ^23^ ^]^ Cell's response to the mechanical properties of their microenvironment is well known from 3D cultures of mammalian cells, whose proliferation, migration, or differentiation programs depend on the viscoelasticity^[^ ^24^ ^]^ and the degradation kinetics of the hydrogel network.^[^ ^25^, ^26^ ^]^ Studies in engineered hydrogels with viscoelastic properties that can be modulated by the type of network crosslinks (reversible/dynamic vs permanent) and by the nature of the degradable sequences have helped to understand and quantify eukaryotic organism's mechanosensitivity range and response.^[^ ^27^ ^]^ Preliminary studies from us^[^ ^10^ ^]^ and others^[^ ^21^ ^]^ on hydrogel‐embedded bacteria have indicated that increasing stiffness of the hydrogel network hinders extension of the bacterial cell wall and thus reduces bacterial growth. Based on these observations, we hypothesized that the behavior of encapsulated bacterial colonies, e.g., growth rate or metabolic activity, might be regulated by tuning the viscoelastic properties of the embedding matrix. Hydrogels formed by the triblock copolymer Pluronic F127 (Plu, PEG_106_‐PPO_70_‐PEG_106_)^[^ ^28^ ^]^ and chemically cross‐linkable derivatives,^[^ ^16^, ^23^, ^29^ ^]^ previously used for bacterial encapsulation in ELMs, seemed appropriate as a model system to test this hypothesis. Plu solutions above critical micellar concentration (0.725 wt% at 25 °C^[^ ^30^ ^]^) self‐assemble in water and form micelles.^[^ ^31^ ^]^ At concentrations >5 wt% and temperatures above 14 °C, micelles self‐assemble through physical interactions and form an associative hydrogel (**Figure** **1**a).^[^ ^28^, ^29^, ^31^ ^]^ Such hydrogels swell and dissociate into individual micelles when immersed in water. When Plu is mixed with its diacrylated derivative (PluDA), similar hydrogels are formed but in this case they can be stabilized by covalent cross‐linking of the acrylate end‐groups. By varying the polymer concentration between 5 and 30 wt%, hydrogels with a storage modulus between 1 and 50 kPa can be obtained.^[^ ^31^ ^]^

In this work, we show that by varying the covalent cross‐linking degree in Plu/PluDa hydrogels, we can tune their viscoelastic properties, which in turn influences the behavior of encapsulated *Escherichia coli*. We reveal fundamental insight on bacterial growth rate and metabolic function in response to material's mechanics. Our results provide guidance for the design of functional and safe ELMs, by which the function of the embedded organisms is supported, controlled, and improved.

## 2 Results

### 2.1 Physicochemical Properties of Pluronic F127/Pluronic F127–Diacrylate Mixtures

Pluronic F127‐based hydrogels were formed at a polymer concentration of 30% w/v, commonly used in engineered living materials studies.^[^ ^16^, ^23^, ^29^ ^]^ The Plu/PluDA mixed hydrogels were prepared by mixing 30% w/v solutions of Plu and PluDA in different ratios (0/100, 25/75, 50/50, 75/25, 100/0) at 4 °C. The 30% w/v Plu/PluDA mixtures formed hydrogels at temperatures above 14 °C (Figure S1g, Supporting Information). To covalently crosslink the hydrogels, a light exposure step was used to photoinitiate the radical polymerization of the acrylate groups. This process yielded transparent hydrogels with constant polymer content, similar organization of the physical network (as reflected by the values of the shear modulus before photopolymerization, Figure S1g, Supporting Information), and different degrees of covalent crosslinking.

The combination of physical (reversible) and chemical (permanent) crosslinks confers Plu/PluDA hydrogels (named DA 0 to DA 100 in the following text) viscoelastic properties that vary with PluDA concentration. The hydrogels showed increasing storage modulus (from 18 to 43 kPa) with increasing concentration of PluDA from 0% to 100% (Figure 1b, Table S1 and Figure S2, Supporting Information). Creep‐recovery experiments were performed to estimate the elastic, viscoelastic, and plastic contributions to the mechanical response (Figure S3, Supporting Information). A stress to reach a peak strain of 1% after 3 min was applied to the different hydrogels, and the recovery was monitored for further 3 min. The hydrogels behaved as viscoelastic solids (Figure 1c). The stronger viscous response of hydrogels with high Plu concentration is a consequence of the dynamic nature of a physical network (Figure 1d). The polymer chains and the micelles are associated with reversible interactions and can reorganize under the applied load.^[^ ^32^ ^]^ With increasing PluDA ratio, the hydrogels required a higher load to reach the peak strain and showed higher elastic recovery. The covalent bonds introduced by PluDA fix the position of PluDA chains in a permanent crosslinked network and confer elastic properties to the hydrogel. This is reflected as a decline in the viscoelastic and plastic contributions to the mechanical response (Figure S3, Supporting Information), summed up as the nonelastic response in Figure 1d. In summary, the viscoelastic properties of the DA0‐100 hydrogels can be tuned depending on the extent of covalent crosslinks incorporated.

### 2.2 Colony Growth and Metabolic Activity inside Pluronic Hydrogels with Different Ratios of Chemical Crosslinking

**Figure 2:**
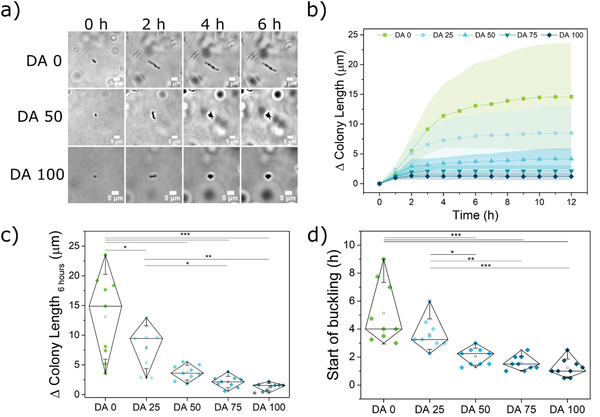
Bacterial growth inside DA 0‐100 hydrogels inserted in microchannels, and quantification of geometrical and dimensional features of the growing colonies. a) Time lapse bright‐field images of bacterial colonies within DA 0, DA 50, and DA 100 hydrogels (scale = 5 µm) representing the differences in the morphology of the growing colonies. b) Increase in the in plane length of the encapsulated bacterial colonies along the longitudinal axis of the first cell with time (mean ± standard deviation). c) Increase in the length of individual colonies at 6 h timepoint. d) Time point for the first buckling event along the longitudinal axis of a growing bacterial colony in the different hydrogels. *N* = 9 colonies from three independent experiments. (Diamond plots indicate 10 and 90 percentile values, whiskers indicate standard deviation values, *p*‐values are calculated using one‐way ANOVA with post hoc Tukey HSD test, * *p* < 0.05, ** *p* < 0.01, *** *p* < 0.001).

Plu/PluDA hydrogels loaded with bacteria in LB medium were injected in microchannels of a commercial microfluidics chip or formed as films in a microwell plate (Figure S4, Supporting Information). In both formats, bacteria were cultured under static conditions, i.e., no medium exchange. The bacteria‐laden hydrogels were transparent to the eye and for microscopy. Bacteria encapsulated within microchannels were imaged by bright field microscopy. Right after encapsulation, isolated bacteria were homogeneously distributed across the hydrogel. Bacteria were not motile inside the hydrogels, presumably due to the physical confinement imposed by the hydrogel network. Bacteria divided inside the hydrogels and daughter cells remained bundled together in what we will hereafter refer to as a “colony.” Initially, progeny cells arranged end‐to‐end, in a chain‐like morphology (**Figure** **2**a–c). As the length of the colony increased, buckling events occurred along the chain of growing cells, wherein 2 adjacent cells deviated from the linear chain and daughter cells emerging from them formed parallel or branched chains on continued growth (Figure 2a,d and Figure S5 and videos, Supporting Information). This was observed as an increase in the in‐plane width (Figure S6b, Supporting Information) of the colonies (i.e., measured in the focal plane of imaging), or an increase in contrast when the buckled cells overlapped in the Z direction. Beyond 6 h, the colony size increased minimally. The rate and extent of colony elongation varied with the ratio of chemical crosslinking in the hydrogel. More pronounced and faster elongation was observed in hydrogels with lower chemical crosslinking ratio (Figure 2b). The overall in plane length reached by the colonies and the time before their first buckling event occurred decreased with increasing ratio of chemical crosslinking (Figure 2a–d). The mean colony length at 6 h dropped from 13.1 ± 7.1 µm for DA 0 to 1.2 ± 0.6 µm for DA 100 (Figure 2c). The first buckling event occurred at a mean time point of 5.1 ± 2.2 h for DA 0 and at 1.2 ± 0.6 h for DA 100 (Figure 2d and Figure S5 and videos, Supporting Information). This time point corresponded to the 4^th^ or 10^th^ division cycles in DA 25 or DA0 gels and to the 1^st^ or 2^nd^ division cycle in DA 100. The growing colonies in hydrogels with higher PluDA ratio showed a more rounded shape. This was reflected in the smaller in‐plane aspect ratio of the colony (Figure 2a and Figure S6c,f, Supporting Information). DA 25, DA 50, and DA 75 showed intermediate behavior with regard to all the above‐mentioned aspects. These results indicate that the covalent crosslinking of the hydrogel matrix impacts the growth behavior of the encapsulated bacteria in the 3D network. The higher the chemical crosslinking degree of the matrix, the slower the bacteria elongation rate, the smaller the size of the colonies (as measured within the focal plane), and the more rounded shape the colonies adopt. Interestingly, the distribution of the in‐plane colony length values and of the buckling times narrowed as the ratio of chemical crosslinks in the hydrogel increased, i.e., the permanent crosslinks homogenized the morphology of the individual colonies.

A number of control experiments were performed to assess the impact of nutrients in the observed behavior of encapsulated bacteria. No differences in the diffusion rate of small molecules across the hydrogels with a different crosslinking degree were observed in FRAP experiments (Figure S7, Supporting Information). Growth experiments performed in hydrogels with a twofold nutrient concentration showed a similar shape of the growth curves and a higher in‐plane growth length in DA0 and DA25 hydrogels (Figure S8, Supporting Information). These results suggest that confinement is the dominant factor regulating growth behavior of *E. coli* in hydrogels with higher chemical crosslinking degree (DA 50–100), whereas bacterial growth in hydrogels with lower chemical crosslinking degrees is also influenced by the nutrient concentration under our experimental conditions. Overall, our data confirm our hypothesis that bacterial growth in hydrogels can be regulated by tuning the design of the encapsulating network, more specifically through the type and degree of crosslinking.

**Figure 3:**
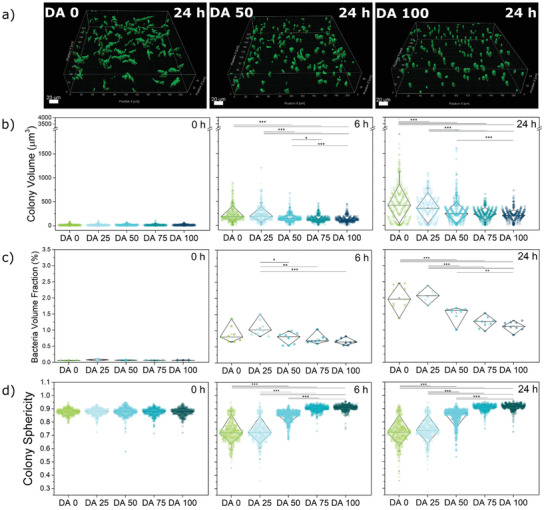
Quantification of bacterial growth in 3D within DA 0‐100 matrices formed in microwells. a) Exemplary volumetric scan of iLOV producing bacterial colonies inside the hydrogels after 24 h. This visualization is presented using surface masks made with the IMARIS software (scale = 20 µm). b) Quantified colony volume within DA 0‐100 hydrogels at 0, 6, and 24 h timepoints. c) Volume fraction of bacterial colonies within DA 0‐100 hydrogels at 0, 6, and 24 h timepoints. d) Sphericity values for individual colonies within DA 0‐100 hydrogels at 0, 6, and 24 h timepoints. (Diamond plots indicate median, 10 and 90 percentile values, whiskers indicate 5 and 95 percentile values, 338 ≥ Number of colonies ≥ 667 from 3 individual experiments, *p*‐values are calculated using one‐way ANOVA with post hoc Tukey HSD test, * *p* < 0.05, ** *p* < 0.01, *** *p* < 0.001).

To better investigate the morphology of the colonies in 3D and quantify colony volumes in the different conditions, we used confocal fluorescence microscopy to follow the colony growth. Experiments were performed in hydrogel films within a microwell plate sealed with silicone oil to prevent evaporation of water from the hydrogel while allowing O_2_ diffusion. Since nuclear staining can cause genetic modifications in the bacteria and impact growth behavior, we opted to use a genetically engineered strain of *E. coli* that constitutively produces the fluorescent iLOV protein.^[^ ^33^ ^]^ This strain grows only slightly slower than unmodified *E. coli* (Figure S9, Supporting Information), presumably as a consequence of the metabolic burden of expressing the protein. Z‐stack images of the bacteria in the hydrogels were acquired at 0, 3, 6, and 24 h as shown in **Figure** **3** and Figure S10 (Supporting Information). Under these conditions, isolated bacteria at 0 h grew into colonies of similar morphologies as observed with unmodified *E. coli* in the microchannels (Figure 2a). Bacteria in hydrogels with higher chemical crosslinking degrees formed smaller colonies, in agreement with our observations in the in‐plane colony length extension analysis (Figure 2), with mean colony volumes ranging from 472 ± 439 µm^3^ for DA 0 to 213 ± 94 µm^3^ for DA 100 (Figure 3b). Higher chemical crosslinking degrees restricted the maximum size the colonies could reach. The volumes of the larger colonies (95^th^ percentile) drop from 1115 µm^3^ in DA 0 to 359 µm^3^ in DA 100 (Figure 3b). Colony volume values were more homogenous (narrower distribution) in gels with higher degrees of chemical crosslinking (Figure 3b). At the whole culture level, the volume fraction of the bacteria within the gels increased with time and decreased with DA content (from 0.06% at the start to 2% for DA 0 and to 1.1% for DA 100 by 24 h) (Figure 3c, Figure S10, Supporting Information). The morphology of the colonies imaged in 3D was also different across the hydrogels. The sphericity of the colonies increased with the degree of chemical cross‐linking (Figure 3d and Figure S11, Supporting Information), in agreement with the trends previously observed in the in‐plane measurements and aspect ratio (Figure 2 and Figure S6c,f, Supporting Information). The distribution of bacterial volume fraction and colony sphericity was narrower in hydrogels with higher degree of covalent cross‐linking (Figure 3c,d), also in line with the in‐plane measurements (Figure 2). Additional experiments with iLOV producing bacteria in microchannels (Figure S12, Supporting Information) showed similar trends, indicating that the control mechanism of bacterial colony growth and morphologies imparted by the chemical crosslinks (i.e., by the mechanical constraint) is independent of metabolic variations.

**Figure 4:**
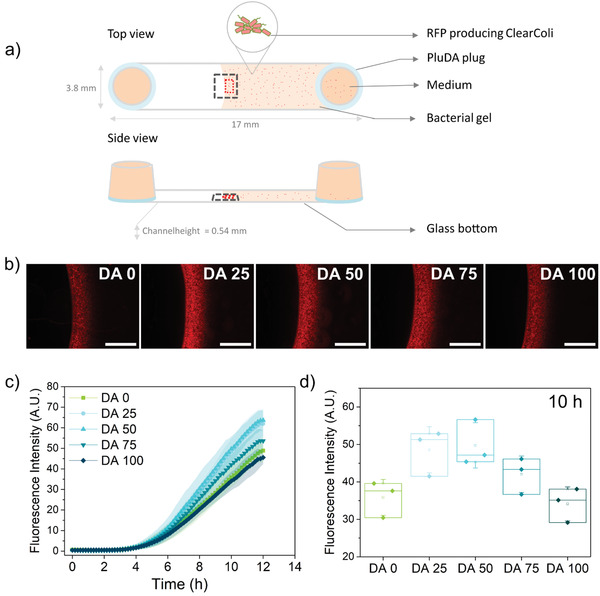
Quantification of protein production by bacteria encapsulated in DA 0‐100 hydrogels. a) Schematic of the bacterial gels within the microchannels with an open end. The black dotted box (3636 × 2727 µm^2^) represents the observed field of view in the microscope, taken at ≈ 0.27 mm height, and the red dotted box (200 × 600 µm^2^) is the area considered for RFP intensity measurement. b) Fluorescence images of RFP producing bacterial gels indicating RFP expressed by the encapsulated bacterial colonies at 10 h (induction, i.e., blue light illumination started at 0 h, scale = 1000 µm). c) Quantification of fluorescence intensity indicating RFP production in the hydrogels during 12 h (mean ± standard deviation). d) RFP production values within different hydrogel compositions at 10 h (*N* = 3, box represents 25 and 75 percentile values, whiskers indicate standard deviation).

We then explored whether the metabolic activity of the encapsulated bacteria was also influenced by the mechanical constraint of the hydrogel network. It is important to note that induced protein overexpression competes with metabolic resources needed for bacterial growth. Strategies to externally regulate these two processes are of utmost relevance in biotechnological production chains to maximize production yield.^[^ ^34^, ^35^, ^36^ ^]^ For our analysis, we used a red fluorescent protein (RFP)‐producing strain, which can be induced by light to drive overexpression of the protein.^[^ ^12^ ^]^ Since our previous results indicated bacterial growth within the first few hours (Figure 2), induction was initiated right after encapsulation. RFP production was detected after 4–5 h and it increased linearly with time in all hydrogels up to 11–12 h, at which time the signal saturated (**Figure** **4**b). Interestingly, the extent of protein production, quantified at 10 h, was higher in hydrogels with intermediate degrees of chemical cross‐linking (DA25 and DA50) (Figure 4c). To check whether this behavior was driven by a confinement‐dependent balance between metabolic activity and growth, a complementary experiment was performed in which encapsulated bacteria were first allowed to grow for 6 h (rapid growth phase) in the dark (no induction) after which protein production was induced by light. Under these conditions, the extent of RFP production did not vary appreciably across the different gel compositions (Figure S13, Supporting Information), even though the gels with higher cross‐linking degrees would have a lower number of cells. This suggests that the slower growing colonies in hydrogels with higher chemical crosslinking were more efficient in RFP production compared to colonies growing faster in physical hydrogels. Thus, bacterial growth restrictions imposed by the mechanical properties of the Plu/PluDA hydrogel network regulated the metabolic activity of the encapsulated cells. We do not expect differences in the accessibility to nutrients or oxygen inside individual colonies because of their small size, typically diameter <30 µm. These results show that hydrogel networks combining reversible and permanent crosslinks can be used as external modulators of the growth and metabolic activity of cells in ELMs.

### 2.3 Correlation between Bacteria Behavior and Mechanical Properties of the Hydrogel

**Figure 5:**
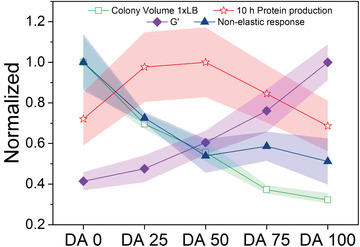
Comparative representation of normalized material's properties and bacterial response as a function of Plu/PluDA matrix composition. Normalized values of shear storage modulus *G*’ and nonelastic response (from Figure 1), 95^th^ percentile volumes of colonies after 24 h (from Figure 3), and protein production after 10 h (from Figure 4). The values were normalized with respect to the highest value in each category (Original values plotted in Figure S14, Supporting Information). The symbols and shaded regions of the normalized colony volume data indicate 95 ± 2 percentile value range, for all other data the symbols and shaded regions indicate mean ± SD.

**Figure** **5** summarizes the trends observed in bacterial behavior and mechanics of Plu/PluDA hydrogels. The extent to which the colony volume grows correlates with the nonelastic component of the mechanical response (Figure 5 and Figure S14, Supporting Information). Protein production showed a more complex dependence of the mechanical properties of the gel, with a biphasic behavior as a function of elasticity. Mechanical restriction seems to favor increased protein production rates, possibly by slowing down growth rates. Beyond a certain level of restriction, the growth is suppressed to such an extent that reduces the overall protein production. A recent report demonstrated that photosynthesis in cyanobacteria can also be regulated by mechanical confinement,^[^ ^37^ ^]^ corroborating that confinement could be used as external factor to regulate metabolic processes in bacteria.

## 3 Discussion

During biofilm formation, soil homeostasis, or invasion of biological tissues, microorganisms grow within confined spaces and push against their natural surroundings to accommodate new cells and grow as colony. The resulting compressive forces on the cell population are dependent on the mechanical properties of the local microenvironment, cell–cell and cell–matrix interactions and mechanical instabilities at cellular scale,^[^ ^38^ ^]^ and have been shown to influence cell size,^[^ ^39^ ^]^ to limit cell growth^[^ ^40^, ^41^ ^]^ and to delay cell cycle^[^ ^42^ ^]^ in studies with microorganisms in synthetic model systems.^[^ ^43^, ^44^ ^]^ Physical models under discussion consider self‐driven jamming and build‐up of large mechanical pressures as natural principles behind the collective growth and organization of a colony in 3D confinement.^[^ ^42^, ^45^ ^]^ Macromolecular crowding and slower diffusion inside the cell, as a consequence of confined growth, have been recently suggested as inherent biophysical feedback routes that can regulate the metabolic behavior of microorganisms in confinement.^[^ ^46^ ^]^ Envelope proteins, motility regulators, and secreted extracellular matrix seem to play a role as sensors and triggers of cellular pathways that convert mechanical inputs in biochemical signals or vice versa.^[^ ^41^, ^47^, ^48^, ^49^, ^50^ ^]^ Understanding how bacterial colonies can be regulated by the embedding natural microenvironment is relevant to combat infections or improve soil fertility, but also to improve the performance of biofilters, microbial fuel cells, and in general ELMs of any kind.^[^ ^1^, ^2^, ^3^ ^]^

Most biophysical studies of mechanomicrobiology in 3D spatial confinement have been performed in microchannels of different dimensions^[^ ^42^ ^]^ or in microdroplets.^[^ ^51^ ^]^ Hydrogel networks are alternative model systems for such studies.^[^ ^21^, ^52^, ^53^ ^]^ In physical hydrogel networks (agarose,^[^ ^12^, ^21^ ^]^ alginate^[^ ^13^ ^]^) formed by reversible crosslinks that can rearrange dynamically and dissipate mechanical stress as bacteria push, bacteria grow into large colonies and eventually outgrow. In contrast, in hydrogels stabilized by covalent crosslinks (e.g., PEGDA,^[^ ^52^ ^]^ polyacrylamide,^[^ ^10^ ^]^ acrylate‐modified Pluronic F127^[^ ^16^, ^29^ ^]^), the bacteria grow as small colonies or remain as single cells over time, presumably as a consequence of increasing compressive forces due to the restricted ability of the surrounding material to dissipate growth‐related stress. The DA 0‐100 hydrogels used in our work contain physical and covalent (i.e., permanent) crosslinks at adjustable ratios and yield encapsulating materials with tunable viscoelasticity within a range that allows tuning the behavior of embedded growing *E. coli* populations from a non‐restricted to a restricted mode. In these hydrogels, bacteria form compact, viable and metabolically active colonies of smaller sizes and at slower rates when the ratio of permanent crosslinks increases. Rod‐shaped bacteria exert longitudinal forces during growth, contributed by their turgor pressure (30–300 kPa for *E. coli*) and limited by the rate of cell wall synthesis.^[^ ^21^ ^]^ The stress applied to a surrounding hydrogel has been estimated around ≈10 kPa for *E. coli* at their ends.^[^ ^54^ ^]^ Our results suggest that this stress is enough to deform the physically crosslinked matrix in Plu hydrogels, where bacteria form large colonies and can outgrow if nutrients are available.^[^ ^28^ ^]^ However, Plu/PluDA hydrogels with PluDA fraction >25% restrict bacterial growth and colony size, independent of nutrient availability, in a way that seems to correlate with their higher mechanical resistance to deformation (Figure 5). The growth restriction in turn influences the metabolism of the bacteria, leading to faster‐induced protein overexpression. Notably, the tunability of the 30% w/v Plu/PluDA hydrogels by varying only the ratio of the components affects both the elastic and viscoelastic response of the final gel, which limits the current study from unambiguously determining the influence of specific mechanical components on bacterial behavior. A possible strategy to overcome this limitation in the following studies will be to identify Plu/PluDA hydrogel compositions having different polymer concentrations^[^ ^31^ ^]^ that yield the same storage moduli but have different viscoelastic properties based on the degree of permanent crosslinks.

The ability of hydrogels with permanent crosslinks to restrict the size of the bacterial colonies allows for biocontainment of the bacterial population inside the hydrogel matrix, a highly desired feature in ELMs and related devices.^[^ ^55^ ^]^ 25–30 wt% Plu based hydrogels have been used to encapsulate bacteria (*Escherichia coli*)^[^ ^29^ ^]^ and yeast (*Saccharomyces cerevisiae*)^[^ ^16^, ^17^ ^]^ in ELMs, and maintain bacteria viability for months.^[^ ^16^ ^]^ Such Plu/PluDA based hydrogels are stable in vitro for days^[^ ^23^, ^56^ ^]^ to years^[^ ^16^ ^]^ and in vivo for weeks^[^ ^57^ ^]^ to months.^[^ ^58^ ^]^ Although the specific mechanisms underlying bacterial mechano‐regulation in our study remain to be elucidated, the Plu/PluDA compositions studied in this article can be used to optimize the functionality of bacteria‐based ELMs.

## 4 Conclusion

This study explores the growth and protein production of encapsulated bacteria using DA0‐100 hydrogels with tunable viscoelasticity. Our results indicate that bacterial functions like growth and metabolic production can be regulated by engineering the mechanical properties of the Plu/PluDA encapsulating matrix, independently of the access to nutrients. This is relevant for the design and performance optimization of ELMs containing functional organisms.

## 5 Experimental Section

### Preparation of Precursors Solutions

Pluronic diacrylate (PluDA) was synthesized by reaction of Pluronic F127 (Plu) with acryloyl chloride in the presence of triethylamine according to a reported protocol.^[^ ^31^ ^]^ 30% (w/v) Plu and PluDA stock solutions in Milli‐Q water (for rheological measurements) or LB medium (for bacterial growth measurements) containing Irgacure 2959 photoinitiator at 0.2% w/v were prepared and stored at 4 °C and allowing them to form the gel at room temperature for 10 min.^[^ ^59^ ^]^ The composition of DA0 and DA100 hydrogels corresponded to that of the stock solutions of Plu and PluDA, respectively. DA 25, DA 50, and DA 75 hydrogels were prepared by mixing of the stock solutions in ratios as shown in Table S1 (Supporting Information). For the photoinitiated crosslinking of the hydrogels, these were exposed to UV light (365 nm, 6 mW cm^‐2^) using a OmniCure Series 1500 lamp during 60 s (Milli Q water solutions) or 120 s (LB medium solutions).

### Rheological Studies

The rheological properties were measured using a rotational rheometer (DHR3, TA Instruments) using a parallel plate geometry. A 20 mm Peltier plate/UV transparent plate was used as bottom plate and a smooth stainless steel 12 mm disk was used as top plate. The rheometer was equipped with a UV Source (OmniCure, Series 1500, 365 nm, 6 mW cm^‐2^) for illumination of the hydrogel samples between the rheometer plates. Experiments were performed at room temperature unless otherwise mentioned. To avoid drying of the sample by evaporation during testing, a solvent trap was used, and the sample was sealed with silicone oil. A volume of precursor solution of 35 µL and a gap between plates of 300 µm was used for the experiments. All tests were initiated 10 min after loading the sample on the rheometer.

Strain sweeps were conducted from 0.001% to 1000% at a frequency of 1 Hz. Frequency sweeps were conducted from 0.01 to 100 Hz at a constant strain of 0.1% with a controlled temperature of 23 °C. From these experiments, the linear viscoelastic region was identified (Figure S1a–f, Supporting Information). Temperature sweep experiments were carried out from 4 °C to 40 °C at a 5 °C min^‐1^ ramp rate, 1 Hz frequency, and 0.1% strain value (found to be in the linear viscoelastic range). These experiments were used to characterize the gelation temperature of the solutions (Figure S1g, Supporting Information). The gelation point (defined as crossover point of *G*’ and *G*’’) in the temperature sweep experiment was 15.5 °C for 30% DA 0 solutions and 14 °C for DA 100. Time sweep experiments to monitor the changes in the mechanical properties of the hydrogels during crosslinking were performed. For this purpose, samples were irradiated at 365 nm (6 mW cm^‐2^) during 60 s at room temperature, and the changes in the shear moduli were recorded for a total of 7 min (Figure S2, Supporting Information). All time sweep experiments were performed at least in triplicate.

### Creep Recovery Experiments

Creep‐recovery experiments at peak strain of 1% after 3 min were performed for Plu/PluDA hydrogels. The applied shear stress was monitored during the creep phase and for further 3 min after removal of the shear stress (recovery phase). The contributions of the elastic, viscoelastic and plastic phases were quantified from the recovery phase (Figure S3, Supporting Information).^[^ ^60^ ^]^ The viscoelastic and plastic contributions are summed up as nonelastic contributions.

### Bacteria Cultures

An endotoxin‐free strain of *E. coli* (ClearColi BL21(DE3), BioCat)^[^ ^12^ ^]^ was used for the bacterial growth studies. It was transformed with the plasmid pUC19 to enable Ampicillin resistance and minimize the risk of contamination in the culture. Bacterial cultures were grown for 16 h at 35 °C, 180 rpm in LB Miller medium supplemented with 50 µg mL^‐1^ of Ampicillin to an optical density at 600 nm wavelength (OD600) value between 0.5 and 1. For the fluorescence‐based experiments, we used a previously optogenetically engineered strain of ClearColi that produces red fluorescence protein (RFP) when illuminated with blue light,^[^ ^12^ ^]^ and a ClearColi strain that constitutively produces iLOV protein. The iLOVf gene insert was amplified from the pET28‐iLOVf plasmid (Addgene #63723) and assembled into the pLp‐3050sNuc vector^[^ ^61^ ^]^ (Addgene #122030) using Gibson Assembly. The iLOVf gene is under the constitutive expression of the P48 promoter.^[^ ^62^ ^]^ The construct was sequence verified and subsequently transformed in the Clearcoli BL21 DE3 strain and maintained with the additional supplementation of 200 µg mL^‐1^ Erythromycin. Since these bacteria grew slightly slower than the pUC19 harboring variants (Figure S9, Supporting Information), they were initially cultured for 16 h at 35 °C, 220 rpm in LB Miller medium until an optical density at 600 nm wavelength (OD600) value of 0.2–1 was reached. The RFP producing strain harbors the plasmid pDawn‐RFP (pDnSR) that encodes blue‐light activatable gene expression and provides kanamycin resistance. The red fluorescence signal was used to image bacterial growth inside the hydrogels and to quantify protein production in hydrogel‐encapsulated bacterial populations. The bacteria were cultured for 16 h at 35 °C, 220 rpm in LB Miller medium supplemented with 50 µg mL^‐1^ of Kanamycin to an optical density at 600 nm wavelength (OD600) value between 0.5 and 1.5. All procedures with the light‐inducible bacterial strain were performed either in the dark or under a laminar hood with an orange film that cuts off blue light.

### Bacterial Encapsulation in Hydrogels

Hydrogels with 30% (w/v) polymer concentration were prepared by mixing stock solutions of Plu and PluDA precursors in bacterial medium with bacterial suspensions (at OD_600_ of 0.5–4 × 10^7^ cells mL^‐1^) at 9/1 (v/v) ratio to achieve a final OD600 of 0.05 within the gels. This concentration allowed individual bacteria to be homogeneously dispersed in the hydrogels and most to grow into individual bacterial colonies that did not coalescence during 24 h. This allowed quantification of individual colony dimensions and morphologies throughout the hydrogel. The solutions were stored in ice before and after mixing. At this temperature, Pluronic solutions are liquid and can be homogeneously mixed with the bacteria by pipetting at the start of the experiment.^[^ ^45^ ^]^ Hydrogels were produced in two different formats: i) Inside channels using Ibidi µ‐Slides VI 0.4 (17 × 3.8 × 0.4 mm^3^) or Ibidi µ‐Slide VI 0.5 Glass Bottom (17 × 3.8 × 0.54 mm^3^) consisting of six parallel microfluidic channels and ii) inside ibidi µ‐Slide angiogenesis microwells with polymer coverslip bottom (Figure S4, Supporting Information). For the in‐plane colony length extension analysis (Figure 2a), Ibidi µ‐Slides VI 0.4 were used. 30 µL of this pluronic/bacteria suspension was pipetted into the microchannel, placed on ice, filling the entire channel. For the high magnification confocal microscopy experiments to determine the colony volume, 30 µL of this pluronic/iLOV‐producing bacteria suspension was pipetted into the Ibidi µ‐Slide VI 0.5 Glass Bottom microchannel (Figure S12, Supporting Information). Experiments in Figure 3a were done with Pluronic/iLOV‐producing bacteria suspensions (10 µL) pipetted into the micro‐well of the µ‐Slide angiogenesis dish. Measurements were done near the center of the wells and 50 µm from the bottom. Ibidi µ‐Slides VI 0.5 with glass bottom were used for the experiments with pluronic/RFP‐producing bacteria suspension (Figure 4). 20 µL suspension was pipetted into the microchannel and filled up to half the length of the channel, resulting in a sharp hydrogel‐air interface (Figure 4), to enable oxygen availability required for folding of the RFP protein.^[^ ^12^ ^]^ RFP production was measured 50 µm from the edge of the gel–air interface using a 200 × 600 µm^2^ area as the region of interest. The reservoir at both the ends of the channel was plugged with 20 µL of DA 100 gel and 50 µL of medium to avoid drying of the hydrogel in the channel for all the experiments with channels. The microculture was kept at room temperature for 10 min for physically cross‐linked gelation to occur and exposed for 120 s to a UV Lamp inside Alpha Innotech FluorChem Q system (Biozym, Oldendorf, Germany) (6 mW cm^‐2^) which was the illumination step used to initiate the photopolymerization of the acrylate groups and covalent crosslinking of the hydrogels. This illumination mildly affects bacterial growth rates and protein production (Figure S15, Supporting Information). All hydrogel compositions were illuminated with the same intensity and for the same period of time irrespective of the acrylate content. The bacterial gels in µ‐Slide angiogenesis microwells were topped with 20 µL of silicone oil (350 cSt, Sigma‐Aldrich) to prevent drying of the hydrogel during the experiment.

### Imaging and Quantification of In‐Plane Length Extension of Colonies inside the Channels

Brightfield microscopy analysis was performed using Nikon Ti‐Eclipse (Nikon Instruments Europe B.V., Germany) microscope with 20 × S Plan Fluor objective with a numerical aperture of 0.45 and a working distance of 8 mm, Sola SE 365 II (Lumencor Inc., Beaverton, USA) solid‐state illumination device and an Andor Clara (Andor Technology Ltd, Belfast, UK) CCD camera for detection. Ibidi µ‐Slides VI loaded with the bacterial hydrogels were incubated at 37 °C and 5% CO_2_ using an Okolab (Okolab SRL, Pozzuoli, Italy) incubation chamber coupled to the microscope. Imaging locations were selected near the middle of the channel length and about halfway between the bottom and top of the channel. Such a position was chosen to minimize the possibility of variations in material properties (swelling, dissolution) or nutrient variability that might occur due to diffusion of the medium at the reservoirs. Changes in material properties of the gel near the reservoirs were inferred from the movement of the bacterial gel in and out of the imaging field during the experiment. Time‐lapse imaging was performed from 30 min to 1 h after introducing the bacterial gels in the channel slides and subsequently in the incubation chamber to ensure that the samples reach 37 °C. Time‐lapse imaging was done with an interval of 15 min for 12 h. Each type of experiment was performed in triplicates.

### Quantification of Colony Growth

The pluronic/iLOV‐producing bacteria samples in the ibidi µ‐Slide angiogenesis micro‐wells were imaged by Zeiss LSM 880 confocal laser scanning microscopy (CLSM) at 0, 3, 6, and 24 h timepoints. Two‐photon laser was used for imaging the iLOV protein producing bacterial colonies.^[^ ^63^ ^]^ The exposure conditions were optimized for minimizing cell photodamage using the objective LD C‐Apochromat 40×/1.1 W Korr M27, detection wavelength 499–624 nm, laser wavelength of 880 nm, and power of 5%. Z‐stacks of 50.102 µm were taken in steps of 0.65 µm. Images of a size of (*xy*) 212.5 × 212.5 µm were acquired with a resolution 512 × 512 pixels, twofold line averaging, and using constant values for laser power (5%) and pixel dwell time of 2.06 µs. The digital gain value was 800 for 0, 3, and 6 h measurements and adjusted to 730 for 24 h measurements, as some pixels reached oversaturation. Imaris software (Version 9.0, Bitplane, Zurich, Switzerland) was used to process CLSM image z‐stacks to create 3D images using the Imaris surface tool. The surfaces were generated with smooth function set to 0.5 µm, the background threshold to 10 µm and the minimum surface voxels limit to 10. The sphericity was quantified as the ratio between the volumes of the colony to its surface area (Imaris surface tool). Volume fraction of the bacterial colonies covering the hydrogel samples was calculated as the ratio of sum of all colony volumes and the volume of the observed hydrogel field of view. For calculation of single colony volumes, the colonies touching the edges of the field of view and those which coalesced together were avoided. Colonies below the volumes of 5 µm^3^ (which was approximately the volume of single bacterium) were neglected.

### Protein Production in the Hydrogels

Ibidi µ‐Slide VI 0.5 Glass Bottom microfluidic channels loaded with bacterial hydrogels were used in these experiments. The culture was kept in static conditions at 37 °C using an incubation chamber coupled to a BZ‐X800 (Keyence, Osaka, Japan) microscope. For fluorescence imaging to detect the RFP production, filter channel TRITC (BZ‐X Filter OP‐87764, excitation 545/25, emission 605/70) was used. RFP production was activated, at 0 h/6 h, by illuminating the channels with blue light (BZ‐X Filter GFP OP‐87763, excitation 470/40, emission 525/50) pulses of 500 ms every 10 min for 18 h using the 4× objective (Keyence Plan Apochromat, numerical aperture 0.20, working distance 20 mm) of the microscope. The light intensity and exposure settings were optimized for detecting early time‐point generation of fluorescence and following increase in intensity for several hours. Image processing and analyses were performed using Fiji edition of ImageJ (ImageJ Java 1.8.0). Quantification of the fluorescence intensities was done by determining the mean grey value, at a height of ≈0.27 mm (mid‐point of channel thickness) and 50 µm away from the edge (as larger colonies were observed near edge, owing to possible differences in the mechanical properties at the interface with air^[^ ^20^, ^21^ ^]^) within a 200 × 600 µm^2^ area of the gel and subtracting the mean grey value of the background.

### Statistical Analyses

One‐way analysis of variance (ANOVA) with post hoc Tukey HSD test was performed with results involving more than three data points. Differences were considered statistically significant at * *p* < 0.05, ** *p* < 0.01, *** *p* < 0.001. Analyses were performed with Origin Pro 9.1 software. Concrete parameters used for the different types of analyses performed have been described in the sections and figure captions of the related experiments.

## Conflict of Interest

The authors declare no conflict of interest.

## Supporting information

Supporting Information

Supplemental Video 1

Supplemental Video 2

Supplemental Video 3

Supplemental Video 4

Supplemental Video 5

## Data Availability

The data that support the findings of this study are available from the corresponding author upon reasonable request.
