# Supplementary material for: Regulating Bacterial Behavior within Hydrogels of Tunable Viscoelasticity
Source: Adv Sci (Weinh). 2022 Apr 11;9(17):2106026. doi: 10.1002/advs.202106026 (PMC9189655; doi:10.1002/advs.202106026)
Supplement: Supplementary file 1 — Supporting Information [file ADVS-9-2106026-s004.pdf]

ADVANCED SCIENCE
Open Access

Supporting Information

for *Adv. Sci.*, DOI 10.1002/advs.202106026

Regulating Bacterial Behavior within Hydrogels of Tunable Viscoelasticity

Shardul Bhusari, Shrikrishnan Sankaran and Aránzazu del Campo**

Supporting Information

Regulating bacterial behavior within hydrogels of tunable viscoelasticity

Shardul Bhusari, Shrikrishnan Sankaran, Aránzazu del Campo**

Synthesis of Pluronic diacrylate (Plu-DA)

Dry Plu-DA (20 g, 1.59 mmol) and triethylamine (0.55 ml, 3.98 mmol) dissolved in 200 ml of dichloromethane were charged in a 500-ml round-bottomed flask. Acryloyl chloride (0.25 ml, 6.36 mmol) was added dropwise. The reaction mixture was stirred at 4 °C for 12 h and then at room temperature for 12 h. A white precipitate formed and was filtered. The solution was concentrated and filtered again. Precipitation in diethylether and drying in vacuum for 1 day rendered 18 g of Pluronic-DA. The chemical structure of Plu-DA and the extent of acrylation were determined by ^{1}H-NMR. The acrylation degree was determined by the ratio of the terminal acrylic protons (5.98-6.52 ppm) to the methylene groups of the backbone (1.05-1.28 ppm). Plu-DA with substitution degree of 70% was obtained.

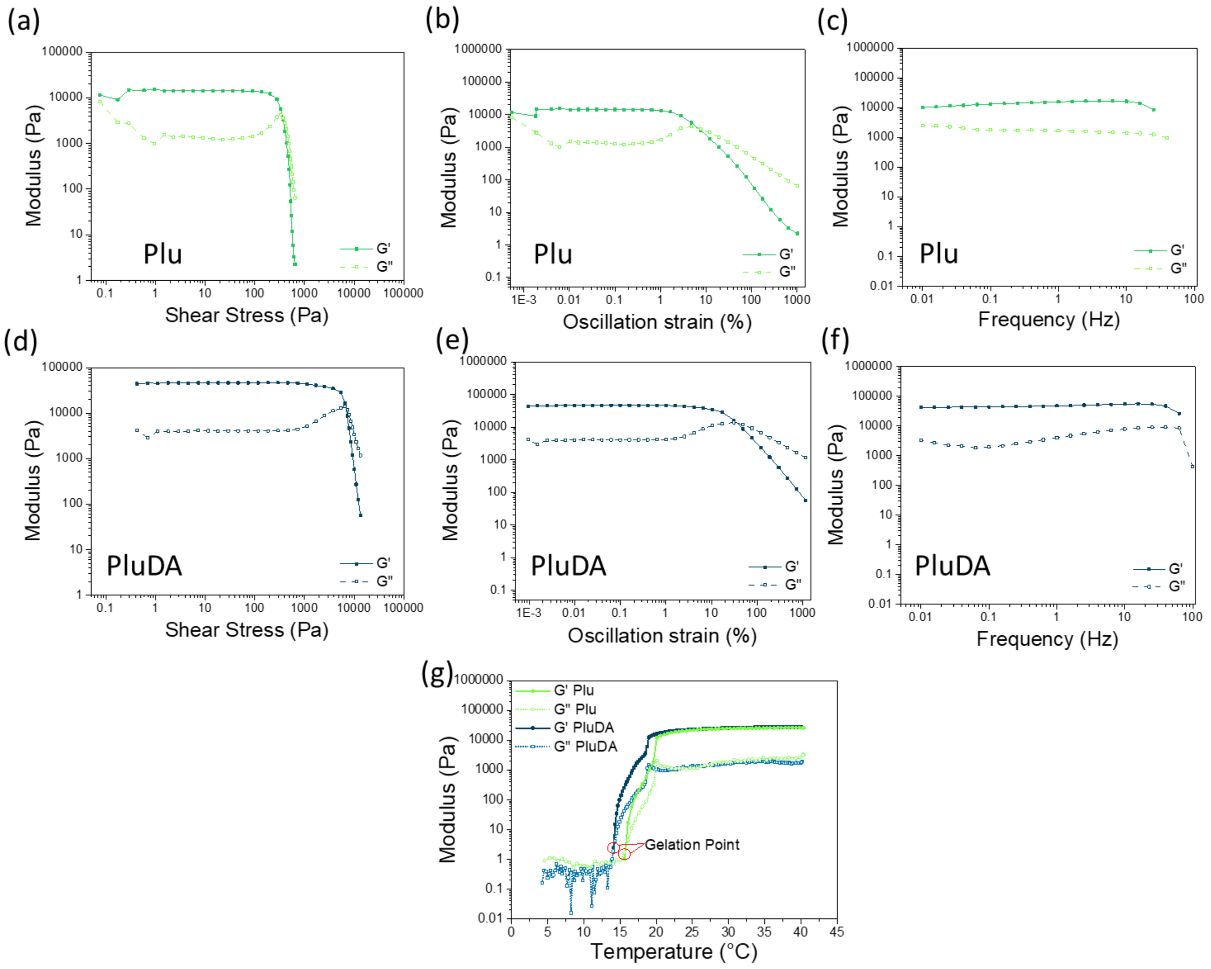

Figure S1. Rheological measurements of Plu and PluDA hydrogels at 30% w/v polymer concentration. **a)** Strain sweep experiment to identify the yield stress value of Plu hydrogel, **b)** Strain sweep and **c)** frequency sweep experiments to determine the linear viscoelasticity region of Plu hydrogel. **d)** Strain sweep experiment to identify the yield stress value of PluDA hydrogel, **e)** Strain sweep and **f)** frequency sweep rheograms to determine the linear viscoelasticity region of PluDA hydrogel after UV irradiation. **a-f**: All measurements were performed at room temperature. **g)** Temperature sweep measurement to determine the gelation point (G'= G'') of Plu and PluDA without UV irradiation.

Table S1. Mechanical properties of DA 0-100 hydrogels with different compositions obtained from rheology: shear storage modulus (G') of the crosslinked hydrogels (Figure 1b, t = 420 s), applied stress in the creep experiment to reach 1% deformation in 3 min (Figure 1c), and the residual deformation (irreversible strain) after recovery (Figure 1c, t = 360 s).

**Hydrogel**	**Composition Plu : PluDA**	**Shear storage modulus [kPa]**	**Applied stress in creep experiment (Pa)**	**Irreversible strain [%]**
DA 0	100 : 0	17.8 ± 1.4	50	0.47 ± 0.22
DA 25	75 : 25	20.4 ± 2.4	100	0.22 ± 0.01
DA 50	50 : 50	25.9 ± 1.5	140	0.18 ± 0.05
DA 75	25 : 75	32.7± 3.8	200	0.29 ± 0.06
DA 100	0 : 100	42.9 ± 1.9	300	0.20 ± 0.13

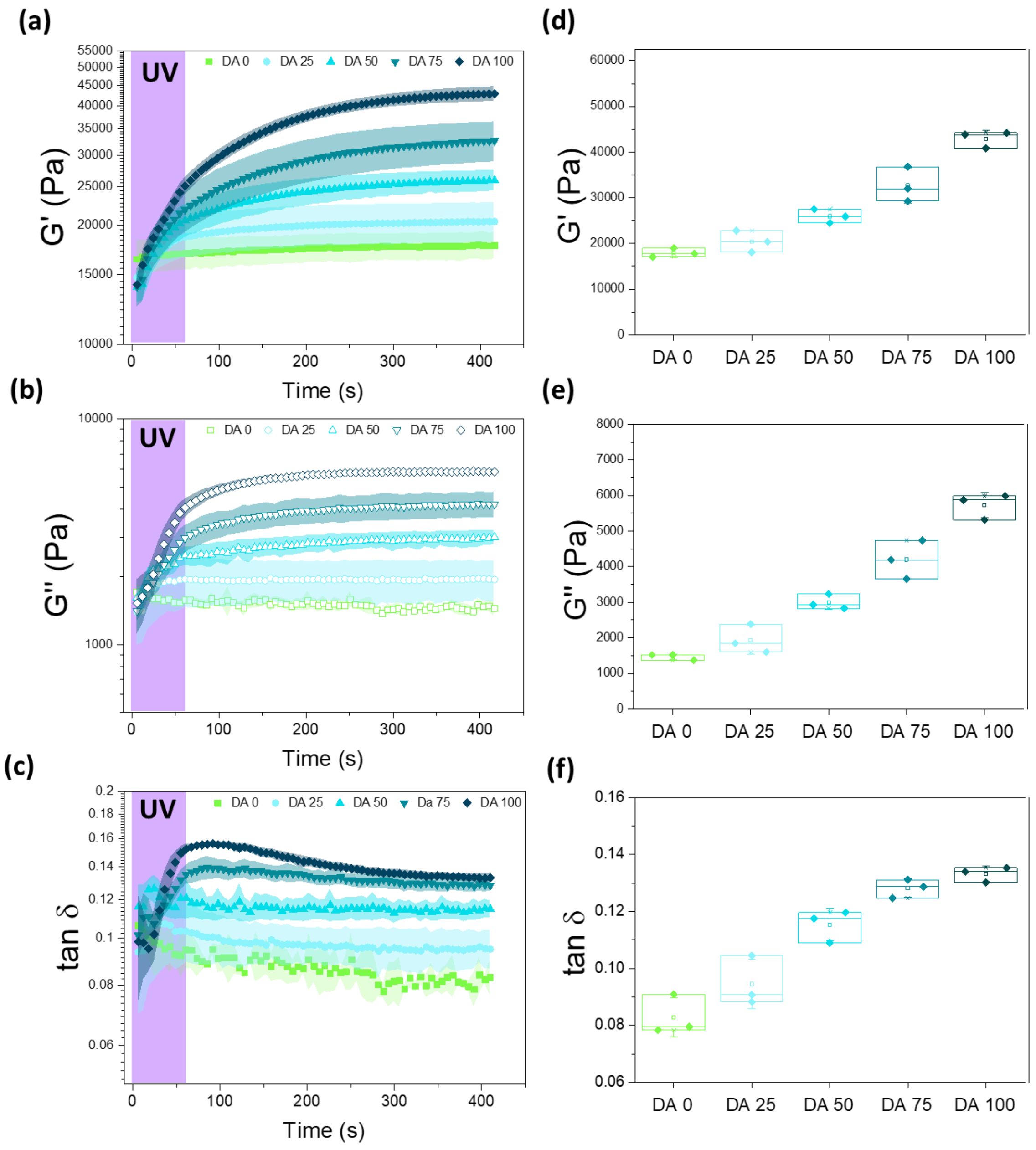

Figure S2. Rheological properties of Plu/PluDA (DA 0-100) hydrogels. **a-c)** Evolution of the **a)** shear storage modulus, **b)** shear loss modulus and **c)** tan δ values after physical gel formation (t=0 s), during photoinitiated crosslinking (t=0-60 s) and afterwards (t=60-400 s). The curves show post-exposure polymerization characteristic of crosslinking processes as a consequence of the hindered mobility of the initiated radicals that slows down the polymerization and termination reactions.[1] **d-e)** Values of **d)** shear storage modulus, **e)** shear loss modulus and **e)** tan δ at t = 420 s from the rheological curves in **a-c**. (N = 3, box represents 25 and 75 percentile values and whiskers indicate standard deviation.)

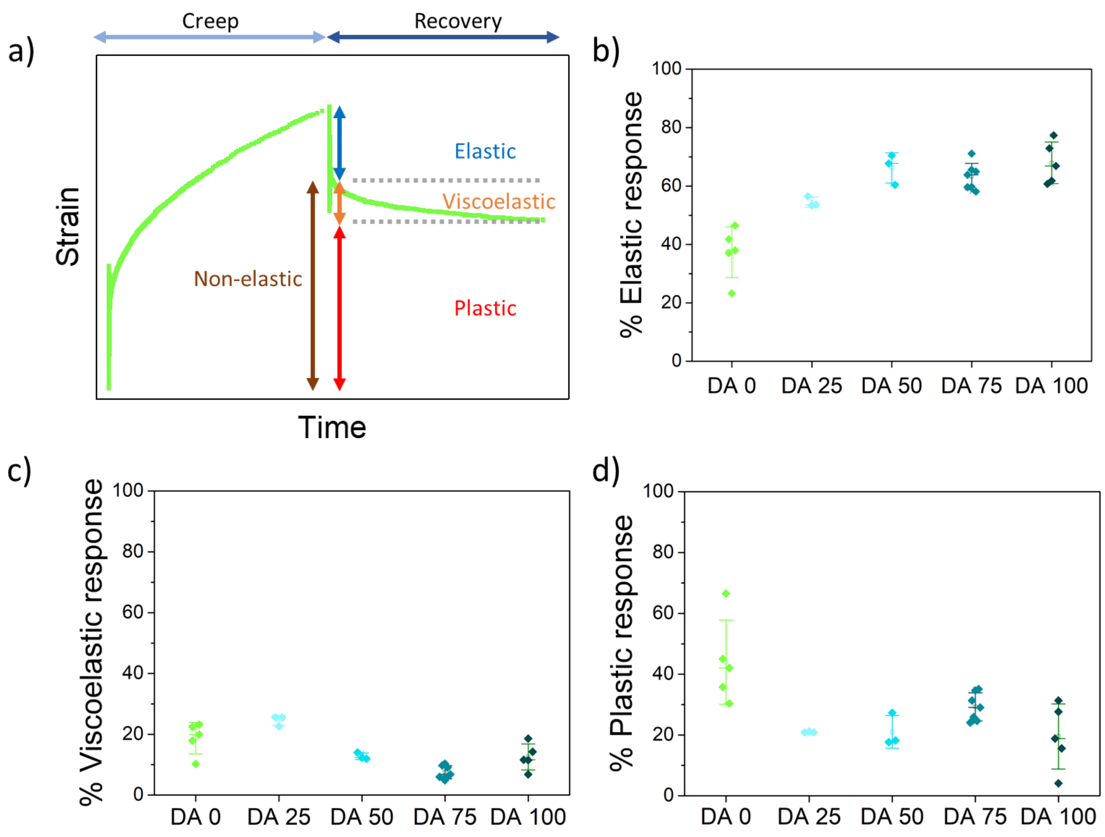

Figure S3. a) The representative creep-recovery curve highlights the basic features of the material response to a constant stress. When a constant stress is applied, the material exhibits instantaneous elastic response and its strain gradually increases over time. A peak strain value is reached at the end of the creep test. After release of the constant stress, the material undergoes elastic and viscoelastic recovery leaving residual or irreversibe strain. b) % Elastic, c) % Viscoelastic and d) % Plastic response of the curves represented in Figure 1c were calculated.

...

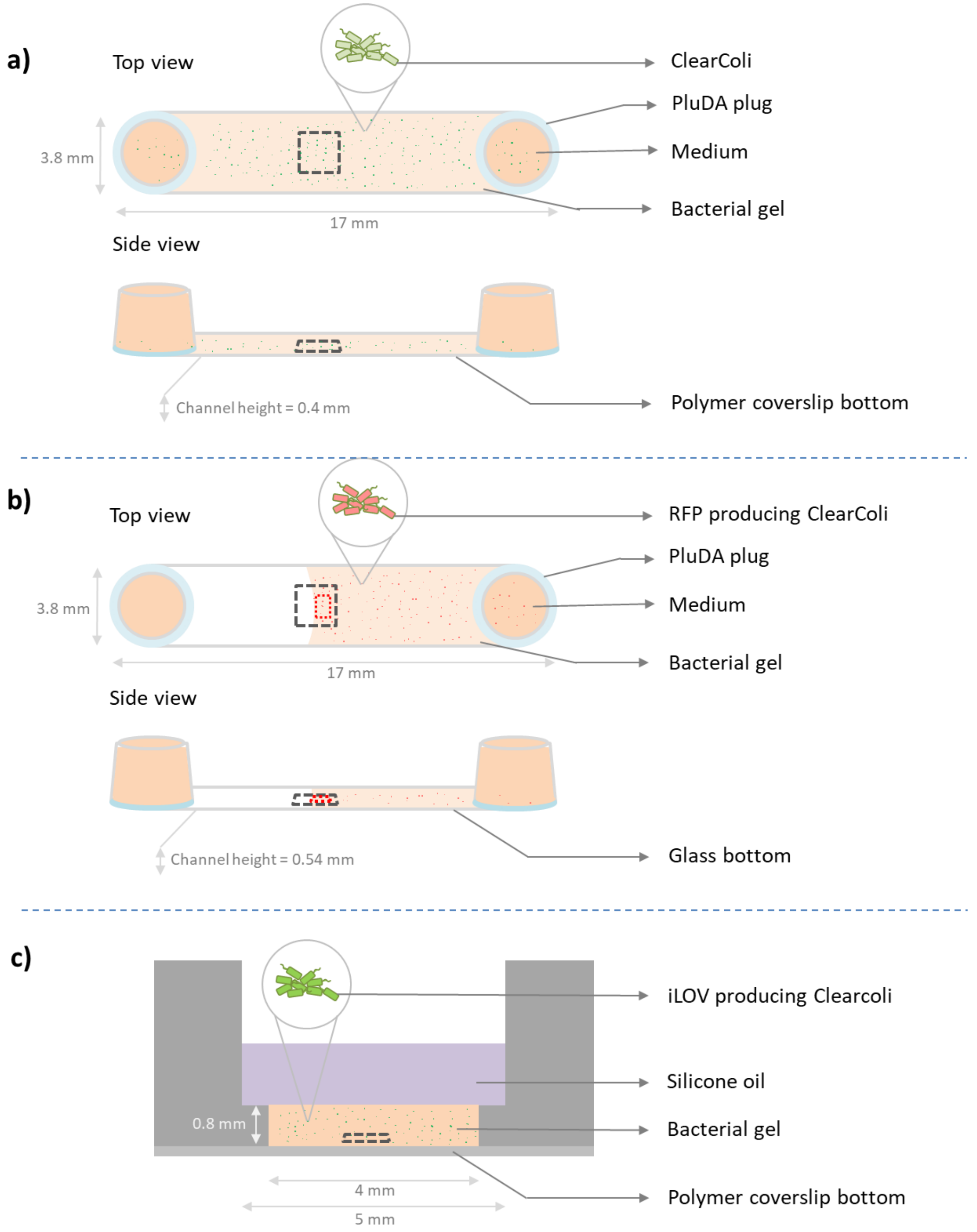

Figure S4. Design of experimental platforms used to quantify bacterial behavior inside the hydrogels. **a)** Microchannels in Ibidi µ-Slides VI 0.4 (17 x 3.8 x 0.4 mm^3). Volume of bacterial hydrogel used to fill the channel was 30 µL, imaging locations (black dotted box of 449 x 335 um^2) were selected near the middle of the channel length and nearly 150-250 µm from the bottom of the channel; **b)** Microchannel in Ibidi µ-Slide VI 0.5 Glass Bottom (17 x 3.8 x 0.54 mm^3). Volume of bacterial hydrogel used to fill the channel was 20 µL, imaging location (black dotted box of 3636 x 2727 $µm^2$) representing the observed field of view in the microscope was selected at the hydrogel-air interface at a height of ca. 0.27 mm (mid-point of channel thickness) and quantification of the fluorescence intensities was done 50 µm away from the edge within a 200 × 600 $µm^2$ area (red dotted box); **c)** Microwell in Ibidi µ- Slide angiogenesis. Volume of bacterial hydrogel used to fill the well was 10 µL, measurement at 50 µm from the bottom (black dotted box of 212.5 × 212.5 × 50.102 $µm^3$). **a-c**) The grey square indicates the region of interest (ROI) of the hydrogel used for imaging, and the red square indicates the region used for quantification of RFP intensity.

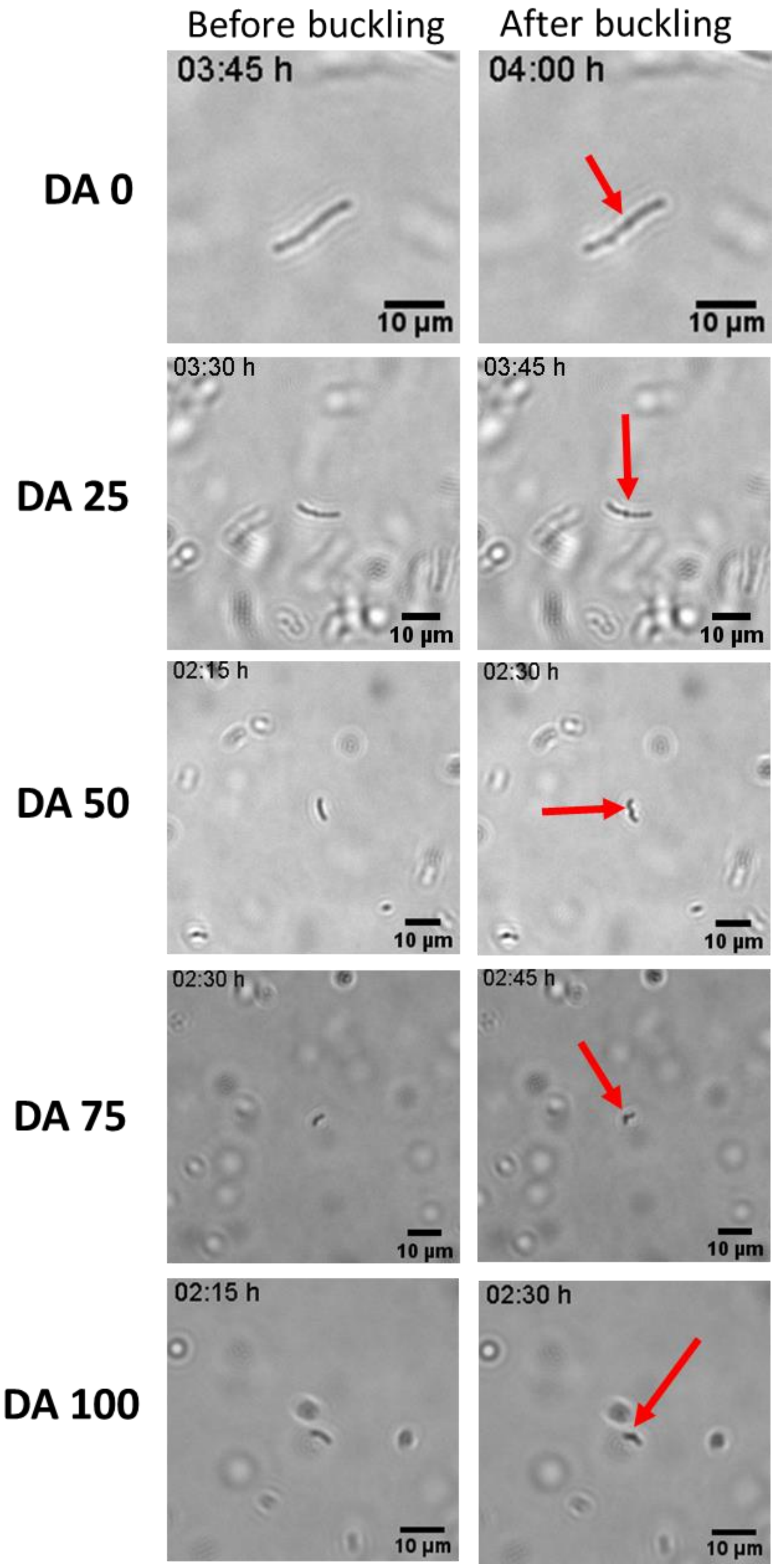

Figure S5. Bacteria colony growth and buckling inside DA0-100 hydrogels in microchannels. Bright field, snap shot images from time lapse video in 1x LB medium. Buckling starts at 4 h in DA0, as appreciated from change in contrast in the middle of the growing bacterial chain (highlighted by red arrows) which is associated to grow in the z plane. Similar phenomena are visible at 2:30 h in DA 100.

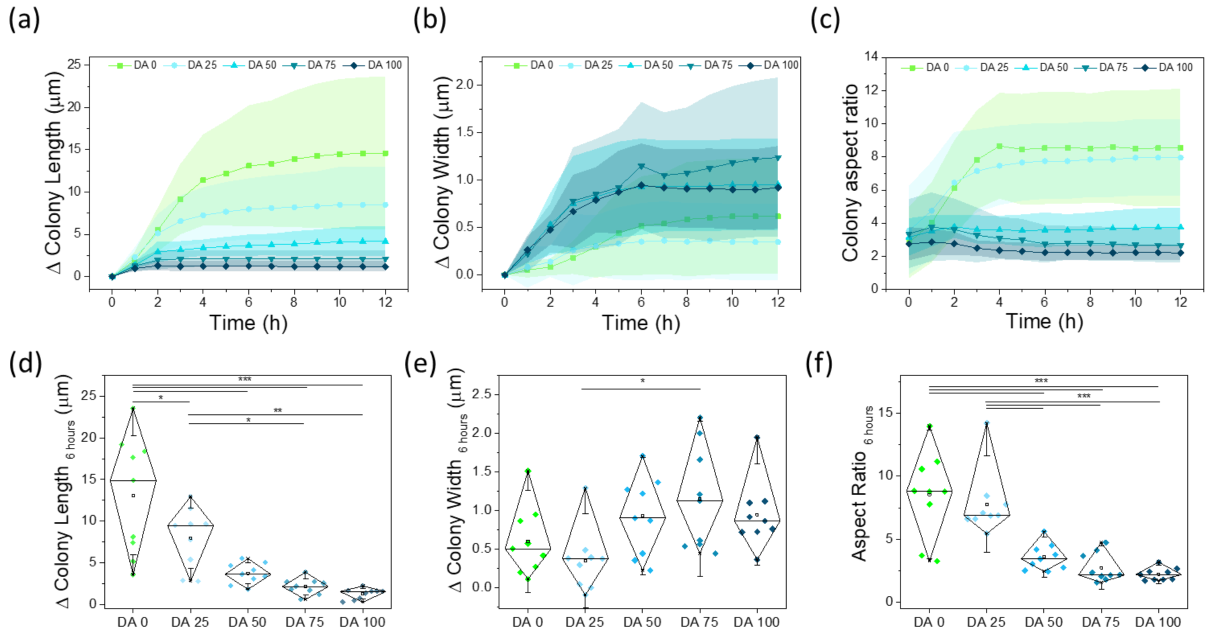

Figure S6. In-plane quantification of dimensional changes of bacterial colonies encapsulated in DA 0-100 hydrogels in microchannels (Figure S4a) with 1x concentration LB medium. **a-c)** Evolution of the length, width and aspect ratio of individual colonies within 12 h of culture (mean ± SD). **d-f)** Comparative values of changes in colony length, width and aspect ratio after 6 hours in the different hydrogels. (Diamond plots indicate 10 and 90 percentile values, whiskers indicate standard deviation values, number of colonies = 9, p-values are calculated using one-way ANOVA with post hoc Tukey HSD test, * $p < 0.05$, ** $p < 0.01$, *** $p < 0.001$)

Fluorescence recovery after photobleaching (FRAP) in the hydrogels

For all of the FRAP measurements, a fluorescent small molecule erythrosin B was used[2] and observed under a confocal laser-scanning microscope (LSM 880) equipped with an Airyscan detector (Zeiss, Germany). The gels, within the microchannel constructs as shown in Figure S4a, were visualized under an air immersion objective (EC Plan-Neofluar 10x/0.30 Ph1 M27), and images were acquired with a 514 nm laser diode, detection wavelength 519-653 nm, dwell time of 0.42 µs per pixel and 0.1% laser power. For bleaching, a circular region of interest with a diameter of 26 µm was illuminated using a 405-nm laser diode at 30% laser power with a dwell time of 131 µs per pixel. The total acquisition length was 261.9 s, with a frame rate of 2 fps.

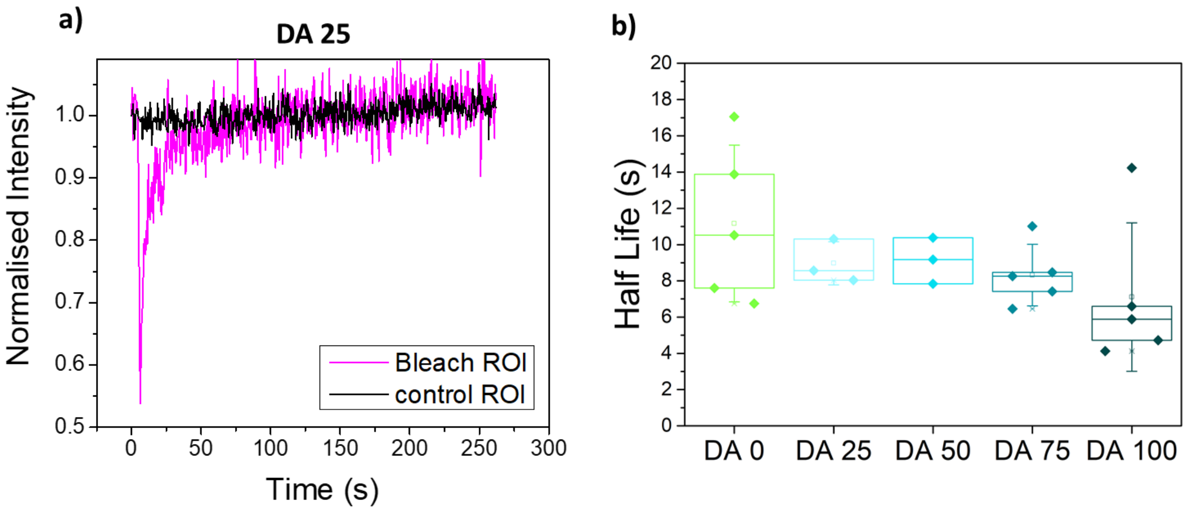

Figure S7. FRAP assay comparison of molecular diffusion rates inside DA 0-100 hydrogels. **a)** Normalized FRAP curve of Erythrosin B dye in DA 25 hydrogel. **b)** Quantified recovery time values, τ ½, of FRAP in the DA 0-100 hydrogels. The obtained τ ½ were close to 10 seconds for all hydrogel compositions with no significant differences. This indicates that the diffusion rate of nutrients within the hydrogels are expected to be similar. ($N \geq 3$, box represents 25 and 75 percentile values and whiskers indicate standard deviation.)

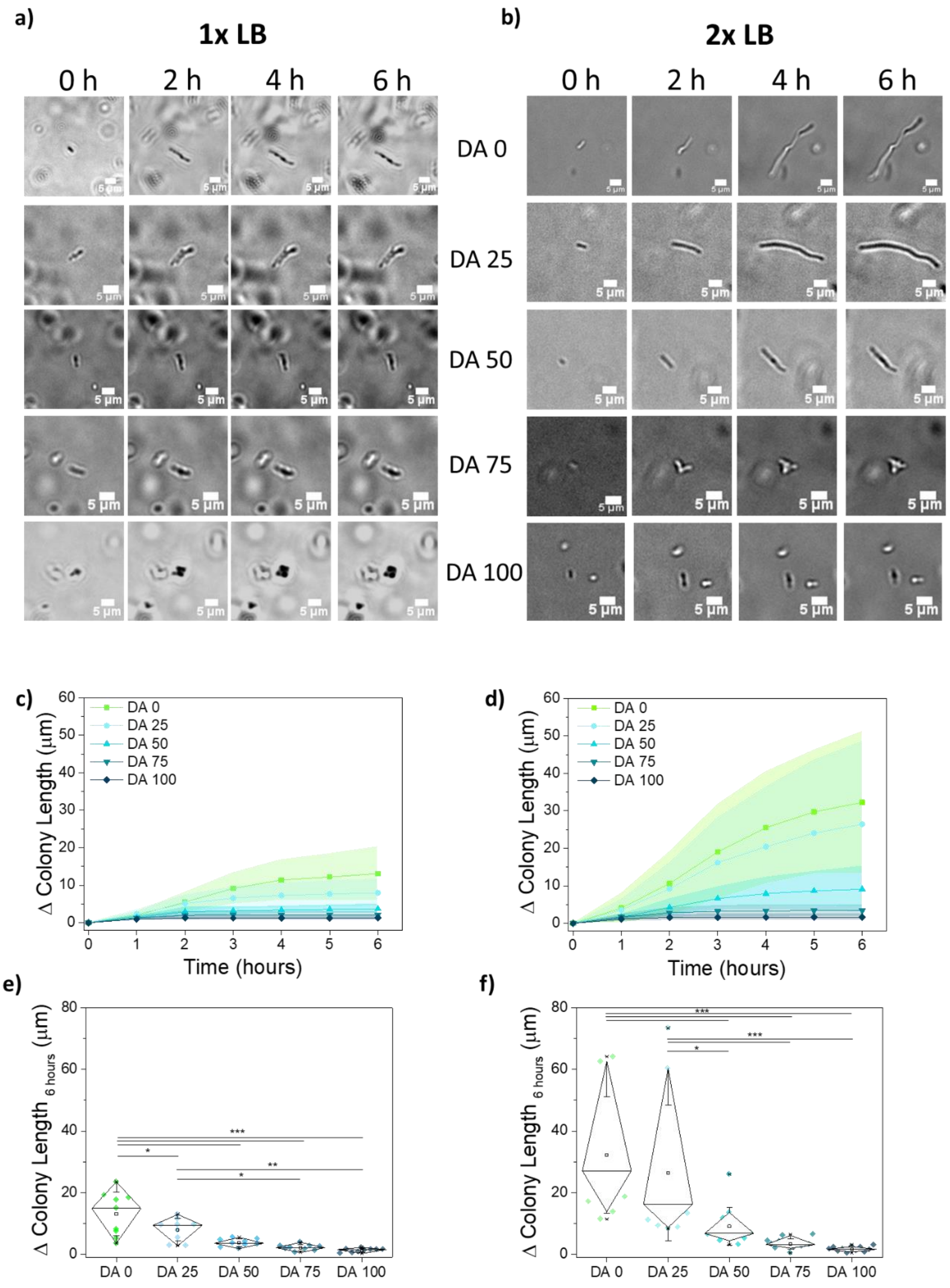

Figure S8. Bacteria colony growth inside hydrogels in microchannels (Figure S4a). **a)** Time lapse bright-field images of bacterial colonies within Da 0-100 hydrogels in 1x LB medium. Scale bar corresponds to 5 µm. **b)** Time lapse bright-field images of bacterial colonies within DA0-100 hydrogels in 2x LB medium. Scale bar corresponds to 5 µm. **c-d)** Quantification of the extension of bacteria colony length within the hydrogels with **c)** 1x and **d)** 2x LB concentration with increasing time; **e,f)** Bacterial length extension values at 6 h with **e)** 1x and **f)** 2x LB concentration. (Diamond plots indicate 10 and 90 percentile values, whiskers

indicate standard deviation values, 9 ≥ Number of colonies ≥ 13, p-values are calculated using one-way ANOVA with post hoc Tukey HSD test, * $p < 0.05$, ** $p < 0.01$, *** $p < 0.001$)

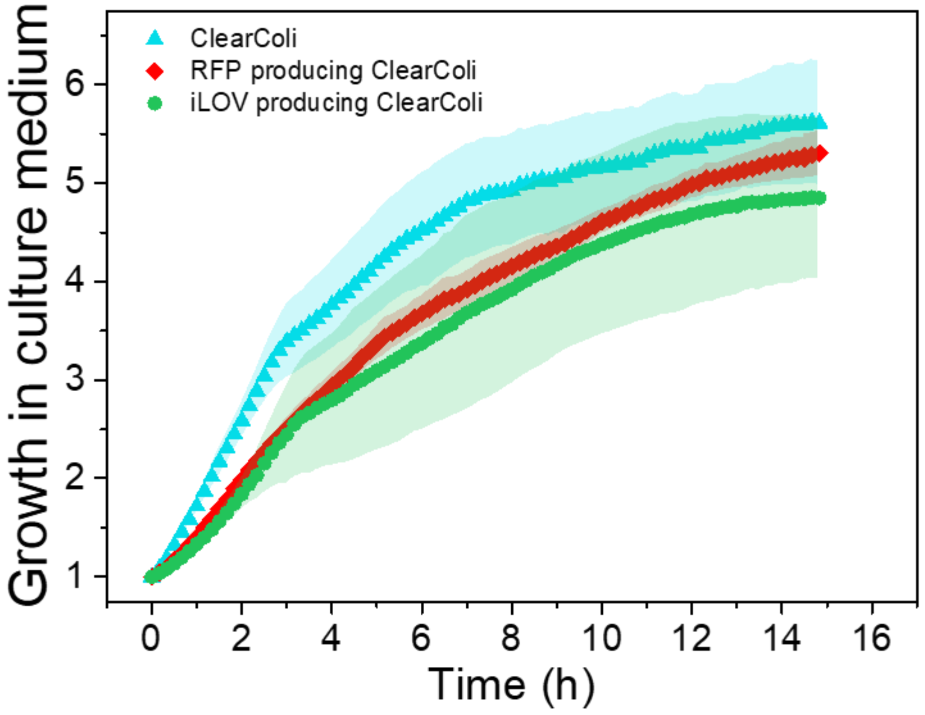

Figure S9. Growth rate of ClearColi, genetically modified RFP producing ClearColi and genetically modified iLOV producing ClearColi bacteria in culture medium determined by OD 600 measurements in a microplate reader (TECAN Infinite 200 Pro) at 37°C with shaking. The y axis indicates the multiplication factor of the growing bacteria population compared to the starting OD 600 of ~ 0.1. The RFP producing ClearColi were pre-irradiated for 5 h under a white lamp before measurement and not further induced during the time course of the experiment. (Symbols – mean, shaded regions – SD, N=3)

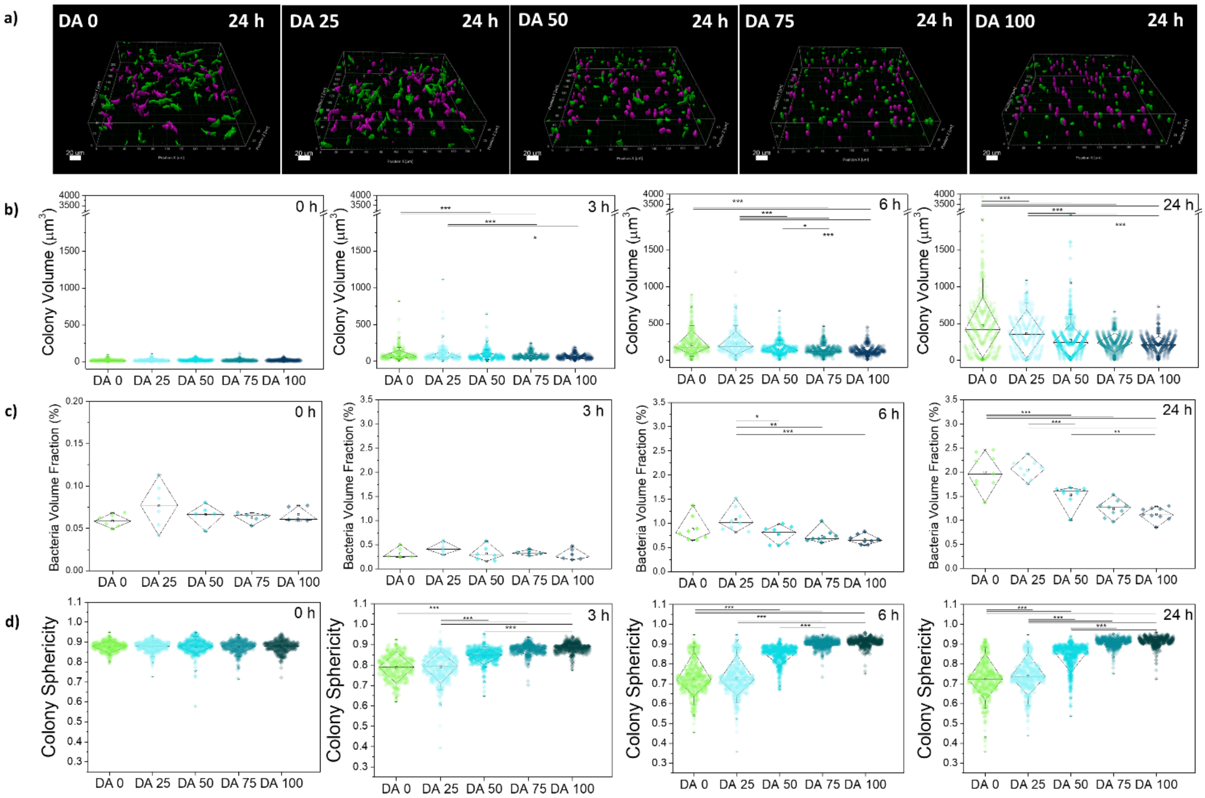

Figure S10. Bacteria colony growth inside hydrogels in microwells (Figure S4c). **a)** Fluorescence image of 212.5 × 212.5 × 50.102 µm^3 volumes of bacterial hydrogels producing iLOV after 24 h culture. The magenta-colored colonies do not touch the edges of the ROI, while the green colored colonies do. The magenta and green colored colonies were used for the quantification of the bacteria volume fraction **(b).** Only magenta-colored colonies were considered for the quantification of the volume **(c)** and sphericity **(d)** of individual colonies. For visualization surface masks obtained by IMARIS software were used. The scale bar corresponds to 20 µm. **b)** Volume of individual colonies in DA 0-100 hydrogels quantified at 0, 3, 6 and 24 h timepoints; **c)** Bacteria volume fraction in DA 0-100 hydrogels quantified at 0, 3, 6 and 24 h timepoints; and **d)** Sphericity of bacteria colonies quantified in DA 0-100 hydrogels. (Diamond plots indicate 10 and 90 percentile values, whiskers indicate 5 and 95 percentile values, 338 ≥ Number of colonies ≥ 667, p-values are calculated using one-way ANOVA with post hoc Tukey HSD test, * $p < 0.05$, ** $p < 0.01$, *** $p < 0.001$)

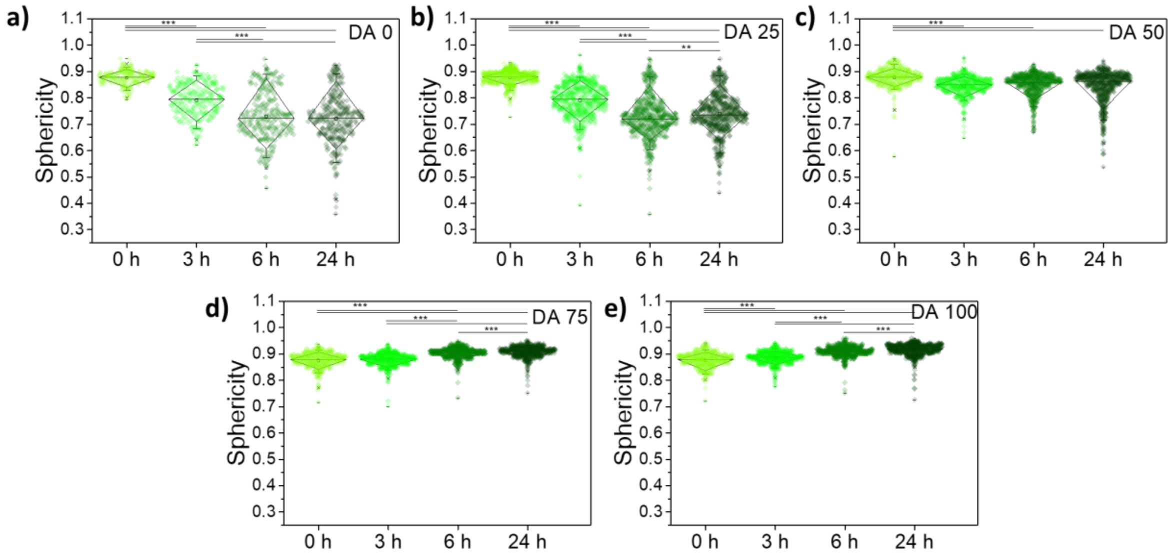

Figure S11. Quantification of the sphericity of iLOV producing ClearColi colonies within DA 0-100 hydrogels, a) DA 0, b) DA 25, c) DA 50, d) DA 75 and e) DA 100 in microwells (Figure S4c) as function of culture time. (Diamond plots indicate 10 and 90 percentile values, whiskers indicate 5 and 95 percentile values, $338 \geq$ number of colonies ≥ 667, p-values are calculated using one-way ANOVA with post hoc Tukey HSD test, * $p < 0.05$, ** $p < 0.01$, *** $p < 0.001$)

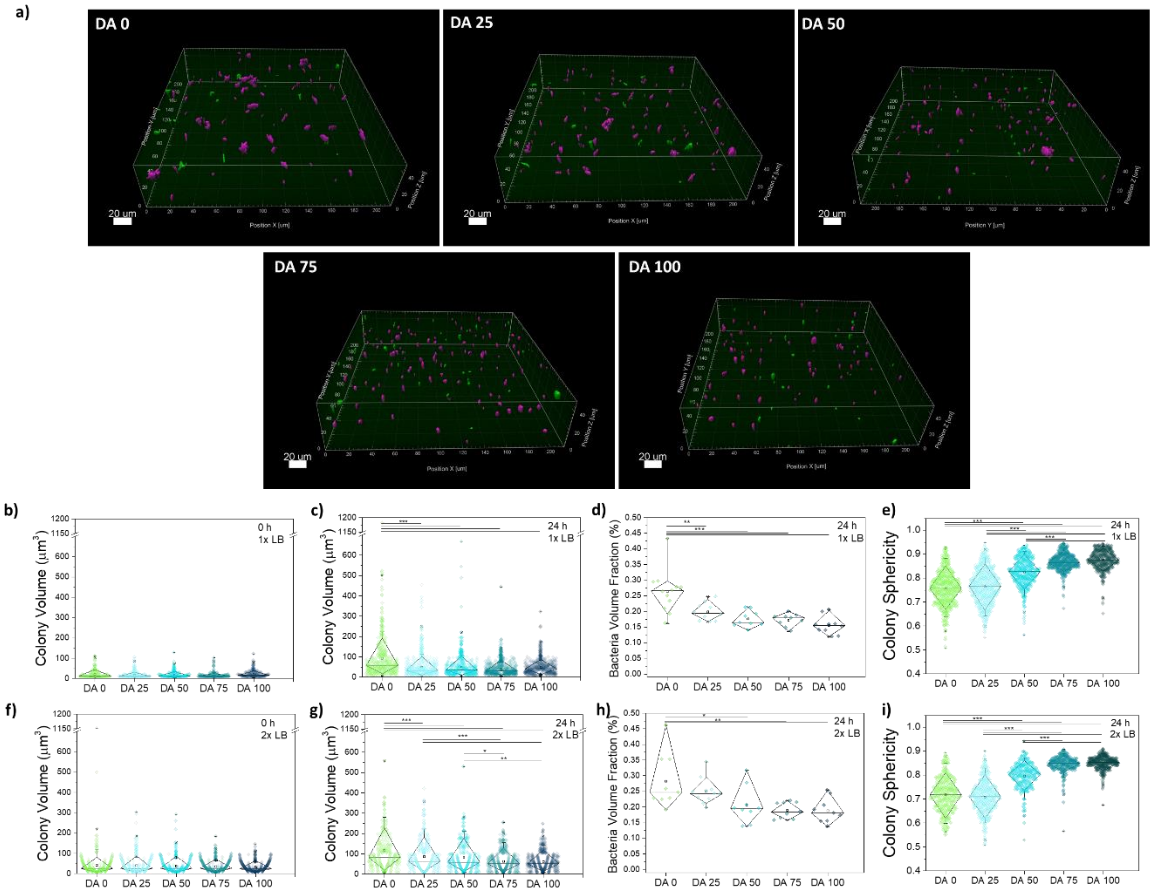

Figure S12. Bacteria colony growth inside hydrogels in microchannels (Figure S4b microchannel, filled with bacterial gel volume of 30 ul). a) Fluorescence image of 212.5 × 212.5 × 50.102 μm^3 volumes of 1x LB concentration bacterial hydrogels producing iLOV after 24 h culture. The magenta-colored colonies do not touch the edges of the ROI, while the green colored colonies do. The magenta and green colored colonies were used for the quantification of the bacteria volume fraction (d,h). Only magenta-colored colonies were considered for the quantification of the volume (b,c,f,g) and sphericity (e,i) of individual colonies. For visualization surface masks obtained by IMARIS software were used. The scale bar corresponds to 20 µm; b) Colony volume distribution within different hydrogels of 1x LB concentration at 0 h and c) 24 h; d) Volume fraction of bacteria colonies within different hydrogels of 1x LB concentration at 24 h timepoint; e) The sphericity of the colonies within different hydrogels of 1x LB concentration at 24 h timepoint and f) Colony volume distribution within different hydrogels of 2x LB concentration at 0 h and g) 24 h; h) Volume fraction of bacteria colonies within different hydrogels of 2x LB concentration at 24 h timepoint and i) The sphericity of the colonies within different hydrogels of 2x LB concentration. (Diamond plots indicating 10 and 90 percentile values, whiskers indicating 5 and 95 percentile values, p-values are calculated using one-way ANOVA with post hoc Tukey HSD test, * $p < 0.05$, ** $p < 0.01$, *** $p < 0.001$)

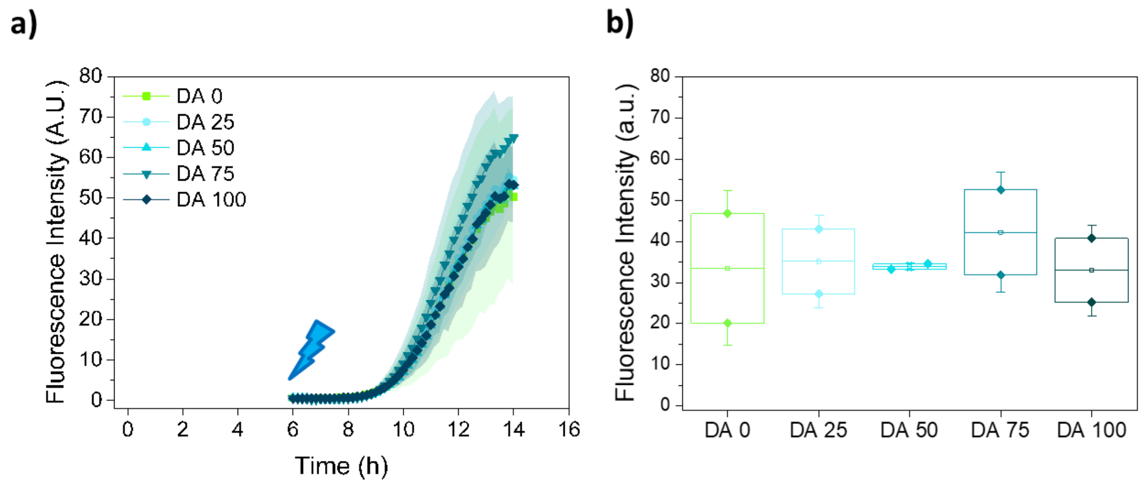

Figure S13. RFP production analysis. **a)** Normalized fluorescence intensity (mean ± standard deviation) associated to RFP production measured in bacteria loaded DA 0-100 hydrogels in microchannels (Figure S4b). The hydrogels were cultured for 6 h and then irradiated to induce protein production. **b)** Fluorescence intensity measured in the hydrogels at 12 h (6 h after the onset of irradiation). (N = 2, box represents 25 and 75 percentile values, whiskers indicate standard deviation)

Different formats lead to different metabolic conditions but result in similar bacterial behavioral trends

When the gel-encapsulated bacteria were allowed to grow in micro-channels, confocal imaging at 0 h and 24 h timepoints indicated that colony volumes increased over time but were smaller (5 – 8x on average) compared to colony volumes in the micro-well format. The trend of colony volumes decreasing and sphericity increasing with increase in chemical cross-linking degree was still observed and was more pronounced when using medium with 2x concentration of nutrients, indicating that the hydrogels were imparting the same type of mechanical constraints in both microwell and micro-channel formats. Another notable aspect was that the fluorescence intensities within the cells were sufficient for quantification at 0 h and 24 h timepoints but not at earlier timepoints (3 h, 6 h), during which the highest rates of bacterial growth are expected to be occurring based on the data from Figure 3.

When RFP-expressing bacteria were encapsulated within the different types of gels and formed within the microchannels with one interface accessible to air, fluorescence appeared only to a depth of ~ 1mm from that interface (**Figure S16**). This indicated that oxygen, which

is required for RFP fluorophore formation, does not diffuse deeper into the gel, causing the bacteria to switch to anaerobic metabolic pathways for growth and sustenance, leading to smaller colony volumes and weaker protein production, as found by previous reports.[3] Such oxygen diffusion limitations are not expected in the hydrogel films in the microwell since the silicone covering film is permeable to oxygen (Figure 3). Nevertheless, even under these conditions, trends of decreasing colony volumes, increasing sphericity and narrowing of distributions for both with increasing chemical cross-linking degrees were similar to the trends observed in the micro-well format. This indicated that the mechanical constraints imparted by the chemical cross-links influenced colony morphologies independent of metabolic variations.

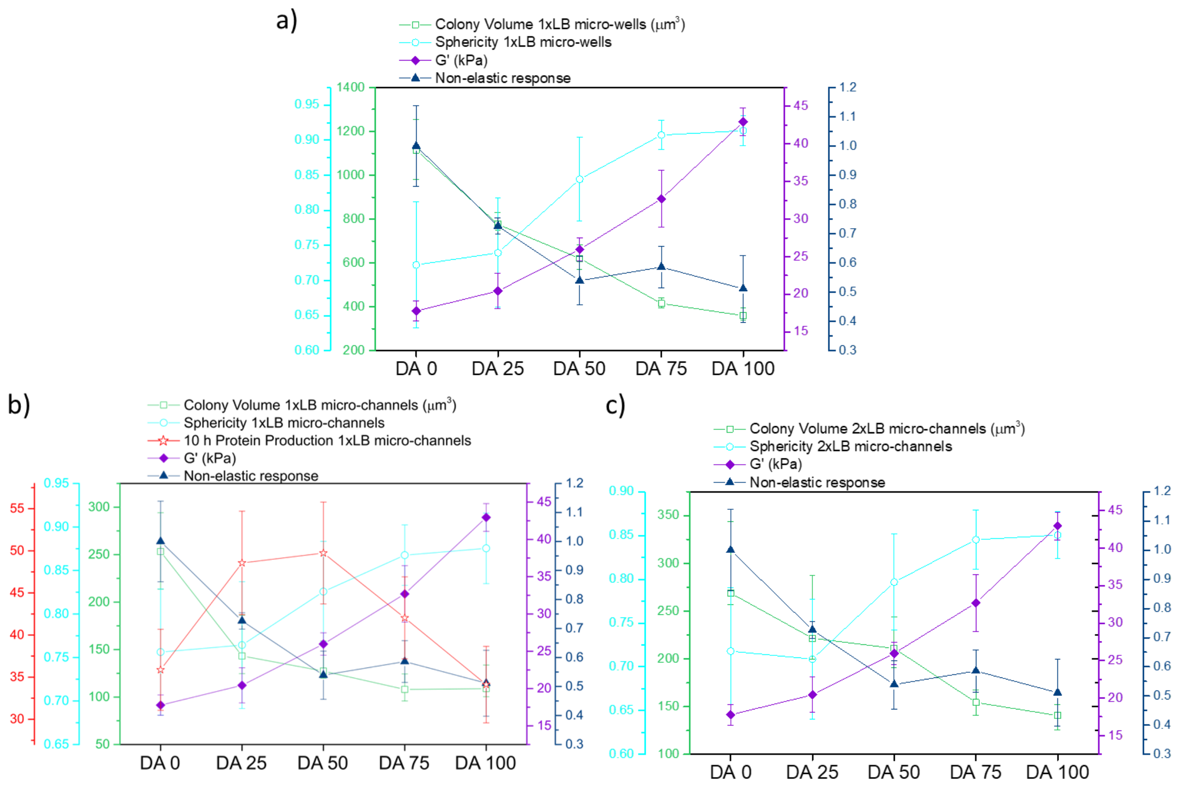

Figure S14. Comparative representation of hydrogel mechanical properties and the behavior of embedded bacteria colonies in DA 0-100 hydrogels in 3 different formats – (a) in micro-wells with 1x nutrient concentration, (b) in micro-channels with 1x nutrient concentration and (c) in micro-channels with 2x nutrient concentration. Mean value of shear storage modulus (from Figure 1b) and proportion of non-elastic response from the creep recovery experiment (from Figure 1d), 95th percentile of colony volume values and mean sphericity after 24 h, and mean protein production after 10 h (from Figure 4d) within the indicated constructs were

plotted. The error bars indicate the 93rd-97th percentile value range for the colony volume data, for the rest, the error bars indicate standard deviation.

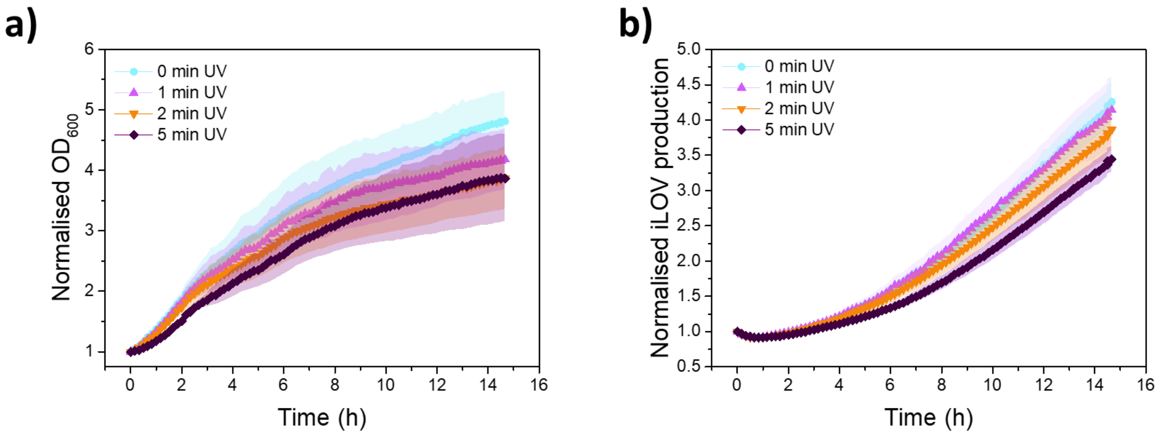

Figure S15. a) Growth rate (OD 600) and **b)** protein production of genetically modified iLOV producing ClearColi in culture medium after UV irradiation (0, 1, 2 and 5 min) determined by OD 600 measurements in a microplate reader (TECAN Infinite 200 Pro, excitation wavelength 447 nm, emission wavelength 497 nm, with gain regulation) at 37°C with shaking. The y axis indicates the multiplication factor of the growing bacteria population compared to the starting OD 600 of ~ 0.1. (Symbols – mean, shaded regions – SD, N ≥ 6).

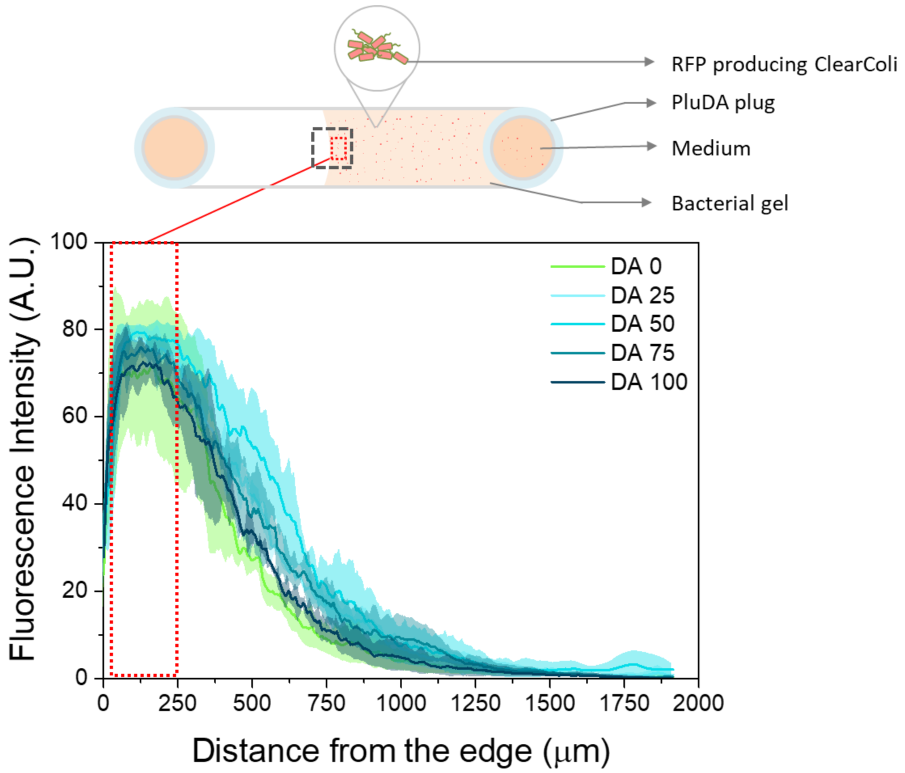

Figure S16. Fluorescence intensity profile in RFP producing bacteria hydrogels inside microchannels (Figure S4b) at t = 18 h. The fluorescence was measured from the edge of the hydrogel to the end of the channel along the channel longitudinal axes. The highlighted dotted

box (200 × 600 μm^3) indicates the region of the bacterial hydrogel considered for RFP fluorescence intensity measurement (Figure 4). (mean ± SD, N=3)

Supplementary video 1 "DA 0_10x video": Bacteria colony growth and buckling inside DA 0 hydrogel in a microchannel.

Supplementary video 2 "DA 25_10x video": Bacteria colony growth and buckling inside DA 25 hydrogel in a microchannel.

Supplementary video 3 "DA 50_10x video": Bacteria colony growth and buckling inside DA 50 hydrogel in a microchannel.

Supplementary video 4 "DA 75_10x video": Bacteria colony growth and buckling inside DA 75 hydrogel in a microchannel.

Supplementary video 5 "DA 100_10x video": Bacteria colony growth and buckling inside DA 100 hydrogel in a microchannel.

References (Supporting Information)

[1] E. Andrzejewska, *Free Radical Photopolymerization of Multifunctional Monomers*, Elsevier Inc., **2016**.

[2] N. B. Hatzigrigoriou, C. D. Papaspyrides, C. Joly, P. Dole, *J. Agric. Food. Chem.* **2010**, *58*, 8667.

[3] J. Von Wulffen, RecogNice-Team, O. Sawodny, R. Feuer, *PLoS ONE* **2016**, *11*, e0158711.
